# Plasma metabolomics of children with aberrant serum lipids and inadequate micronutrient intake

**DOI:** 10.1371/journal.pone.0205899

**Published:** 2018-10-31

**Authors:** Katherine J. Li, NaNet Jenkins, Gary Luckasen, Sangeeta Rao, Elizabeth P. Ryan

**Affiliations:** 1 Department of Environmental and Radiological Health Sciences, College of Veterinary Medicine and Biomedical Sciences, Colorado State University, Fort Collins, Colorado, United States of America; 2 University of Colorado Health Research–Northern Region, Medical Center of the Rockies, Loveland, Colorado, United States of America; 3 Department of Clinical Sciences, Colorado State University, Fort Collins, Colorado, United States of America; University of Hawai'i at Manoa College of Tropical Agriculture and Human Resources, UNITED STATES

## Abstract

Blood lipids have served as key biomarkers for cardiovascular disease (CVD) risk, yet emerging evidence indicates metabolite profiling might reveal a larger repertoire of small molecules that reflect altered metabolism, and which may be associated with early disease risk. Inadequate micronutrient status may also drive or exacerbate CVD risk factors that emerge during childhood. This study aimed to understand relationships between serum lipid levels, the plasma metabolome, and micronutrient status in 38 children (10 ± 0.8 years) at risk for CVD. Serum lipid levels were measured via autoanalyzer and average daily micronutrient intakes were calculated from 3-day food logs. Plasma metabolites were extracted using 80% methanol and analyzed via ultra-high-performance liquid chromatography-tandem mass spectrometry. Spearman’s rank-order coefficients (r_s_) were computed for correlations between the following serum lipids [total cholesterol, low-density lipoprotein (LDL) cholesterol, high-density lipoprotein (HDL) cholesterol, and triglycerides (TG)], 805 plasma metabolites, and 17 essential micronutrients. Serum lipid levels in the children ranged from 128–255 mg/dL for total cholesterol, 67–198 mg/dL for LDL, 31–58 mg/dL for HDL, and 46–197 mg/dL for TG. The majority of children (71 to 100%) had levels lower than the Recommended Daily Allowance for vitamin E, calcium, magnesium, folate, vitamin D, and potassium. For sodium, 76% of children had levels above the Upper Limit of intake. Approximately 30% of the plasma metabolome (235 metabolites) were significantly correlated with serum lipids. As expected, plasma cholesterol was positively correlated with serum total cholesterol (r_s_ = 0.6654; p<0.0001). Additionally, 27 plasma metabolites were strongly correlated with serum TG (r_s_ ≥0.60; p≤0.0001), including alanine and diacylglycerols, which have previously been associated with cardiometabolic and atherosclerotic risk in adults and experimental animals. Plasma metabolite profiling alongside known modifiable risk factors for children merit continued investigation in epidemiological studies to assist with early CVD detection, mitigation, and prevention via lifestyle-based interventions.

## Introduction

Cardiovascular disease (CVD) contributes to more than 17 million adult deaths per year worldwide [1], yet estimating and managing disease risk remains a challenge. Childhood and adolescence has become a common time for the emergence of CVD risk factors, such as aberrant cholesterol and improper dietary habits that persist into adulthood [2]. Chronic imbalances in diet and nutrition are drivers for many metabolic diseases [3]. Elevated serum total or low-density lipoprotein (LDL) cholesterol are valuable clinical predictors of atherosclerosis and CVD risk, though not all individuals who develop CVD present with these established risk factors, and thus complicates early disease detection [4, 5]. Dysregulated lipid and amino acid metabolism have been shown to underlie CVD progression, and several small-molecule metabolites in blood have been associated with obesity in children [6, 7]. Small molecules have also shown relevance to dyslipidemia [5, 8–11], type II diabetes [12, 13], and insulin resistance [14, 15] in adults and/or experimental animals. Blood metabolites may also serve as biomarkers that reflect the pathophysiology of cardiometabolic diseases [8, 16]. Metabolomics has demonstrated potential to identify and phenotype small molecules in blood and serum to reveal subclinical metabolic signatures associated with early signs of disease [14, 17], and offers the opportunity to advance our knowledge of personalized medicine and lifestyle intervention initiatives through the sensitive detection and profiling of metabolic changes, partially occurring in pediatric conditions and emerging chronic diseases [14, 17–20].

The plasma metabolome is an amalgamation of genetic, lifestyle, and environmental products, along with capturing information on short- and long-term dietary factors and nutritional compounds that have relevance to health. The Food and Nutrition Board of the Institute of Medicine has provided Recommended Daily Allowance (RDA) or Adequate Intake (AI) values, so the average daily intake levels of a nutrient required for a child’s health is known and can help inform nutritional adequacy [21]. Conversely, nutrient intakes exceeding the Tolerable Upper Level (UL), as a result of overnutrition, contributes to metabolic disturbances [21]. Multiple micronutrients have central roles in metabolism, and the associated anti-oxidant and anti-inflammatory properties of micronutrients can infer cardioprotection [22]. Adequate intakes of riboflavin and niacin are critical to fuel the electron transport chain, and zinc is a cofactor to hundreds of biological enzymes [23]. Furthermore, folate, vitamin B_12_, and vitamin B_6_ are essential in lowering blood homocysteine, a biomarker linked to coronary heart disease and stroke [23, 24]. Integrating micronutrient status with plasma metabolite signatures of CVD risk in children will be critical to advance upon knowledge of how micronutrient-metabolite associations affect cardiovascular health into adulthood.

Plasma metabolite signatures associated with aberrant cholesterol and micronutrient status in children have not previously been examined and was evaluated herein to identify novel targets for intervention and disease prevention. This study characterized the plasma metabolome of children with aberrant cholesterol and identified correlations between serum lipid levels, micronutrient status, and the plasma metabolite profile that have biological importance. Metabolites may serve as novel, sensitive, and modifiable CVD risk biomarkers and efforts to strengthen clinical predictive profiles of CVD risk in children merit attention in conjunction with the standard serum lipid parameters.

## Materials and methods

### Study population

Children (n = 38; 10 ± 0.8 years old) included in this plasma metabolome analysis had participated in a dietary intervention trial as previously described [25, 26]. All children were healthy, as they had no ongoing medical illnesses and were not taking any medications. The aberrant cholesterol levels were identified from a Healthy Hearts school-based screening program and were considered high risk for developing CVD [25]. Study inclusion criteria for aberrant cholesterol was: total cholesterol ≥180 mg/dL and high-density lipoprotein (HDL) cholesterol <60 mg/dL; LDL ≥100 mg/dL and HDL <60 mg/dL; or non-HDL >100 mg/dL and HDL <60 mg/dL. The University of Colorado Health-North Institutional Review Board (Protocol 13–1263) and the Colorado State University Research Integrity and Compliance Review Office (Protocol 13–4390) approved the study protocol. Prior to enrollment, written informed consent were obtained from guardians and written informed assent from all children. This trial was registered at clinicaltrails.gov under NCT01911390.

### Assessment of self-reported micronutrient status

Average daily micronutrient intakes were analyzed from 3-day food logs using Nutritionist Pro (Version 7.1.0) (Table 1). Each child completed food logs with help from their guardian [25]. Micronutrient status was calculated as a percentage of each respective RDA for 9 to 13 years of age [21, 27–31] (S1 Table) using the formula:

$$\%\;RDA=\frac{Daily\;intake\;of\;X\;micronutrient\;(in\;\mu g\;or\;mg)}{RDA\;for\;X\;micronutrient\;(in\;\mu g\;or\;mg)}$$

**Table 1:** Characteristics of children with aberrant cholesterol^a^.

Characteristic	Males (n = 19)	Females (n = 19)	*P*^c^
**Age (years)**	10 ± 1 (10)	10 ± 1 (10)	**-**
**Family history**^**b**^			
High cholesterol	8 (42%)	8 (42%)	-
High blood pressure	7 (37%)	9 (47%)	-
Overweight	8 (42%)	8 (42%)	-
Diabetes	5 (26%)	5 (26%)	-
Mental illness	6 (32%)	5 (26%)	-
**BMI percentile**^**b**^			0.9876
Underweight	0 (0%)	0 (0%)	-
Healthy weight	8 (42%)	8 (42%)	-
Overweight	5 (26%)	5 (26%)	-
Obese	6 (32%)	6 (32%)	-
**Serum lipids**			
Total cholesterol (mg/dL)	179.3 ± 24.4 (173)	161.1 ± 15.7 (162)	**0.0080**
LDL-cholesterol (mg/dL)	117.8 ± 24.9 (113)	94.4 ± 17.3 (92)	**0.0012**
HDL-cholesterol (mg/dL)	44.5 ± 7.2 (44)	43.4 ± 6.6 (44)	0.7341
Triglycerides (mg/dL)	87.8 ± 34.9 (69)	118.9 ± 40.3 (115)	**0.0268**
**Vitamins**			
Vitamin A (μg/day)	720.1 ± 399.7 (586.2)	888.7 ± 589.5 (679.7)	0.4835
Vitamin C (mg/day)	72.9 ± 73.4 (51.9)	94.7 ± 64.3 (85.0)	0.1290
Vitamin D (μg/day)	4.1 ± 2.4 (4.1)	4.2 ± 2.9 (4.3)	0.8839
Vitamin E (mg/day)	1.3 ± 3.1 (0.5)	2.1 ± 3.2 (0.8)	0.0505
Thiamine (Vitamin B1) (μg/day)	1.1 ± 0.4 (1.1)	1.0 ± 0.6 (1.0)	0.4835
Riboflavin (Vitamin B2) (μg/day)	1.5 ± 0.5 (1.4)	1.4 ± 0.8 (1.5)	0.7261
Niacin (Vitamin B3) (mg/day)	13.6 ± 6.0 (13.0)	14.2 ± 7.1 (13.2)	0.6827
Vitamin B6 (mg/day)	1.0 ± 0.4 (1.0)	1.2 ± 0.6 (0.9)	0.5992
Folate (μg/day)	258.5 ± 110.8 (249.1)	246.3 ± 154.5 (209.1)	0.5019
Vitamin B12 (μg/day)	3.2 ± 1.3 (3.1)	3.0 ± 1.8 (2.6)	0.5207
**Minerals**			
Zinc (mg/day)	6.7 ± 2.2 (6.6)	6.2 ± 3.4 (7.1)	0.7481
Calcium (mg/day)	1078.5 ± 714.5 (887.4)	965.0 ± 281.2 (939.1)	0.8495
Potassium (mg/day)	1593.7 ± 618.2 (1398.3)	1755.0 ± 719.4 (1731.8)	0.4655
Sodium (mg/day)	2656.4 ± 653.6 (2627.1)	2887.1 ± 963.3 (2770.2)	0.5207
Iron (mg/day)	10.9 ± 2.8 (10.7)	10.5 ± 3.2 (10.1)	0.6614
Magnesium (mg/day)	158.3 ± 61.3 (139.6)	162.1 ± 77.3 (156.1)	0.7927
Selenium (μg/day)	62.1 ± 32.8 (56.3)	57.2 ± 34.9 (54.3)	0.7927
**Fruit and vegetable intake**			
Fruits (cups/day)	0.8 ± 0.8 (0.5)	1.1 ± 0.7 (1)	0.0659
Vegetables (cups/day)	0.9 ± 1.1 (0.5)	1.1 ± 0.8 (1)	0.1935

BMI, body mass index; HDL, high-density lipoprotein; LDL, low-density lipoprotein.

^**a**^All values are reported as average ± standard deviation (median), unless otherwise noted.

^**b**^Values are number of children (percentage).

^**c**^Mann-Whitney U-test.

The *X* represents vitamin A, B_1_, B_2_, B_3_, B_6_, B_12_, C, D, E, folate, zinc, calcium, potassium, sodium, iron, magnesium, or selenium. Where applicable, the average daily intake of a micronutrient was also compared to respective UL [27–29].

### Blood sample collection and serum lipid measurement

Plasma metabolomics data and 3-day food logs collected at the beginning of the trial (baseline) were included in the analysis. Fasting blood samples were collected from the children by venipuncture into a 4 mL sodium citrate cell preparation tubes and a serum separation tube (BD Biosciences, Franklin Lakes, NJ). Tubes were centrifuged at 1,500 relative centrifugal force at room temperature for 30 minutes to separate plasma from red and white blood cells. The serum lipid panel comprised total cholesterol, LDL-cholesterol, HDL-cholesterol, and TG, and were analyzed using the Vitros 5600 analyzer [25]. Plasma was aliquoted (0.5 mL) and frozen at -80°C until metabolomics analysis.

### Plasma metabolite extraction and identification

Plasma metabolites were extracted and analyzed through a non-targeted metabolomics platform via ultra-high performance liquid-chromatography tandem mass spectrometry (UPLC-MS/MS) at Metabolon, Inc (Durham, NC) as previously described [32]. Briefly, following sample extraction with 80% methanol, the extract was analyzed by reverse phase UPLC-MS/MS with positive and negative ion mode electrospray ionization and hydrophilic interaction liquid chromatography (HILIC) with negative ion mode electrospray ionization using a Waters ACQUITY UPLC and a Thermo Scientific Q-Exactive high resolution/accurate mass spectrometer interfaced with a heated electrospray ionization (HESI-II) source and Orbitrap mass analyzer operated at 35,000 mass resolution. Extracts were analyzed using acidic positive ion conditions optimized for hydrophilic compounds by gradient eluting from a dedicated C18 column (Waters UPLC BEH C18-2.1x100 mm, 1.7 μm) using water, methanol, 0.05% perfluoropentanoic acid, and 0.1% formic acid, or for hydrophobic compounds by gradient eluting using methanol, acetonitrile, water, 0.05% perfluoropentanoic acid, and 0.01% formic acid. A basic negative ion extract was gradient eluted from a separate C18 column using methanol, water, and 6.5 mM ammonium bicarbonate at pH 8. The remaining extract was analyzed via negative ionization following gradient elution from a HILIC column (Waters UPLC BEH Amide 2.1x150 mm, 1.7 μm) using water, acetonitrile, and 10 mM ammonium formate at pH 10.8. The MS analysis alternated between MS and data-dependent MS^n^ scans using dynamic exclusion (70 to 1000 m/z).

The plasma compounds were confirmed by comparison to an internal library of over 3,300 entries of purified standards or recurrent unknown entities maintained by Metabolon. Metabolites were quantified in terms of relative abundance and a median-scaled relative abundance was calculated for each metabolite by dividing its raw abundance by the median value of the metabolite across the entire dataset [32]. The raw and median-scaled relative abundance of all metabolites identified in plasma are presented in a supplementary file (S1 File).

### Statistical analysis

Non-parametric Mann-Whitney U tests were used to compare levels of serum lipids and micronutrients between males and females (p≤0.05). For correlation analyses between plasma metabolites and serum lipids, non-parametric Spearman’s rank-order coefficients (r_s_) were calculated between serum lipids (total, LDL-, and HDL-cholesterol, and TG) and the median-scaled relative abundance for each identified plasma metabolite (805 total) for all children. Correlation analyses were also completed between plasma metabolites and micronutrients (vitamin A, B_1_, B_2_, B_3_, B_6_, B_12_, C, D, E, folate, zinc, calcium, potassium, sodium, iron, magnesium, and selenium), and between serum lipids and micronutrients. All statistical analyses were performed using GraphPad Prism (Version 7). Following the analysis of all significantly correlated plasma metabolites and serum lipids or micronutrients, a cut-off of r_s_ ≥ ±0.60 was applied to reveal strongest correlations. Student t-tests were performed following log-transformation of metabolites to assess if plasma metabolite levels differed between males and females (p≤0.05).

## Results

### Serum lipid levels and self-reported micronutrient status of children

**Fig 1:**
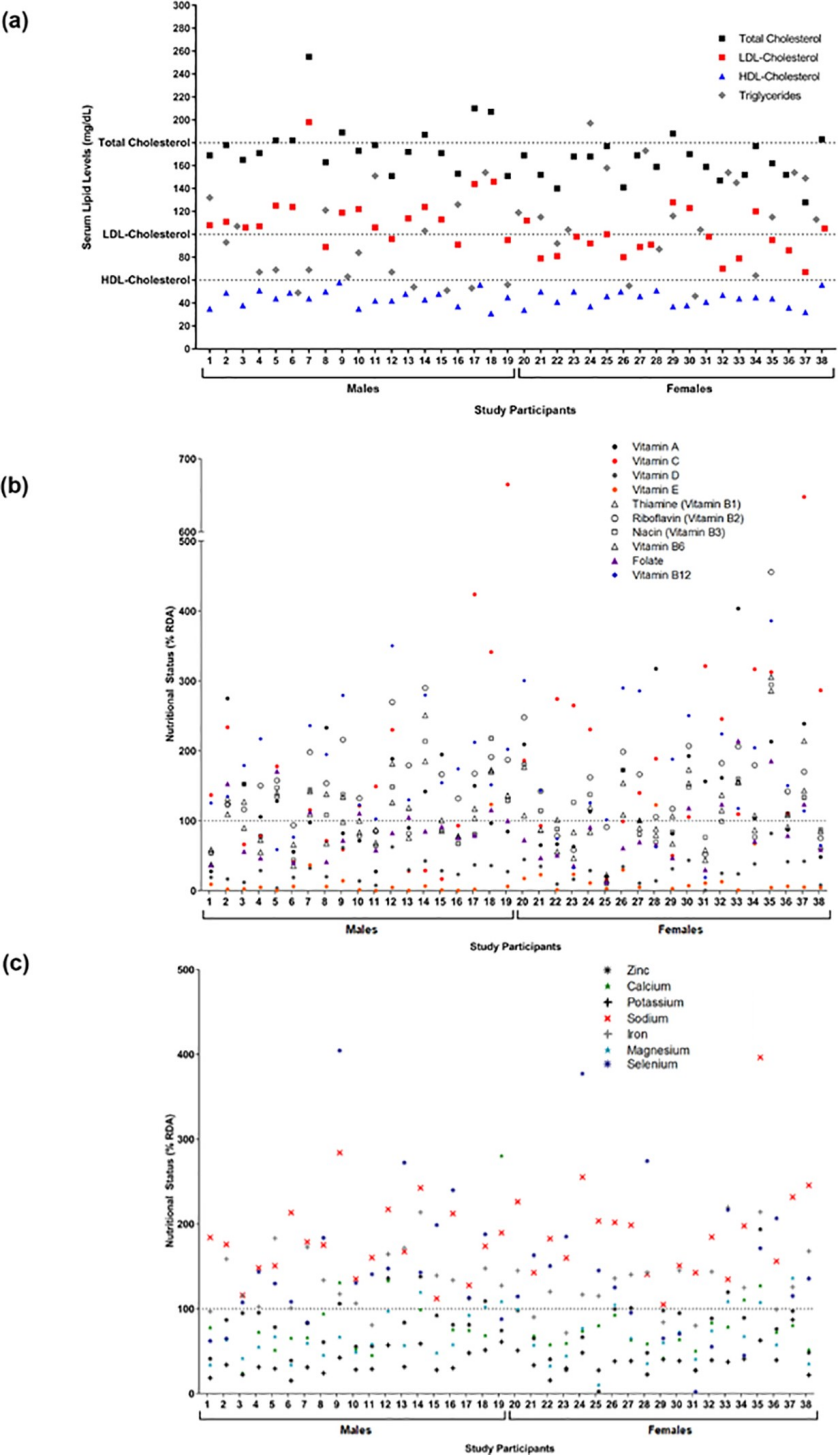
Serum lipid levels and micronutrient status. (a) Levels of total cholesterol, low-density lipoprotein (LDL)-cholesterol, high-density lipoprotein (HDL)-cholesterol, and triglycerides (TG) in all children. Dotted lines represent study inclusion criteria cut-offs for total, LDL-, or HDL-cholesterol. Levels of (b) vitamins or (c) minerals in all children relative to the Recommended Daily Intake (RDA) value for each nutrient. Values above the dotted line indicate that the RDA was met for the micronutrient, and values below the dotted line indicate that the RDA was not met for the micronutrient.

Serum lipids in the 38 children with aberrant cholesterol are presented in Table 1 and Fig 1A. Serum lipids ranged from 128 to 255 mg/dL for total cholesterol, 67 to 198 mg/dL for LDL-cholesterol, 31 to 58 mg/dL for HDL-cholesterol, and 46 to 197 mg/dL for TG (Fig 1A). Males had a higher median total cholesterol (173 vs. 162 mg/dL; p = 0.0080), higher LDL-cholesterol (113 vs. 92 mg/dL; p = 0.0012), and lower TG (69 vs. 115mg/dL; p = 0.0268) compared to females. The HDL-cholesterol levels were comparable between sexes (44 vs. 44 mg/dL; p = 0.7341) (Table 1). Out of the 38 children with aberrant cholesterol, 16 (42%) had reported a family history of high cholesterol, high blood pressure, and/or overweight status. Ten children (26%) had reported family history of diabetes, and 11 (29%) had family history of mental illness (Table 1).

The self-reported micronutrient status of all children is presented in Table 1, including intake information on 10 vitamins and 7 minerals. No significant differences in micronutrient status were observed between males and females. The majority of children in this cohort had adequate vitamin C (68% of children), riboflavin (vitamin B_2_) (79%), vitamin B_12_ (82%), iron (87%), and selenium (74%) levels above the RDA for these micronutrients (Fig 1B and 1C). Adequate levels of vitamin A, thiamine (vitamin B_1_), niacin, vitamin B_6_, and zinc, were achieved by 47, 63, 45, 47, and 37% of children, respectively. There were deficiencies identified in the majority of children for vitamin E (95%), calcium (87%), magnesium (79%), and folate (71%). All children (100%) had vitamin D and potassium levels lower than the RDAs for these micronutrients (Fig 1B and 1C). Furthermore, 76% of children had sodium levels above the UL of 2,200 mg/day [31], 2 children had vitamin A levels above the UL of 1,700 μg/day [29], 4 children had niacin levels above the UL of 20 mg/day [28], 1 child had folate levels above the UL of 600 μg/day [28], 1 child had calcium levels above the UL of 3,000 mg/day [27].

### Plasma metabolite profiles of children with aberrant cholesterol and inadequate micronutrient intake

**Fig 2:**
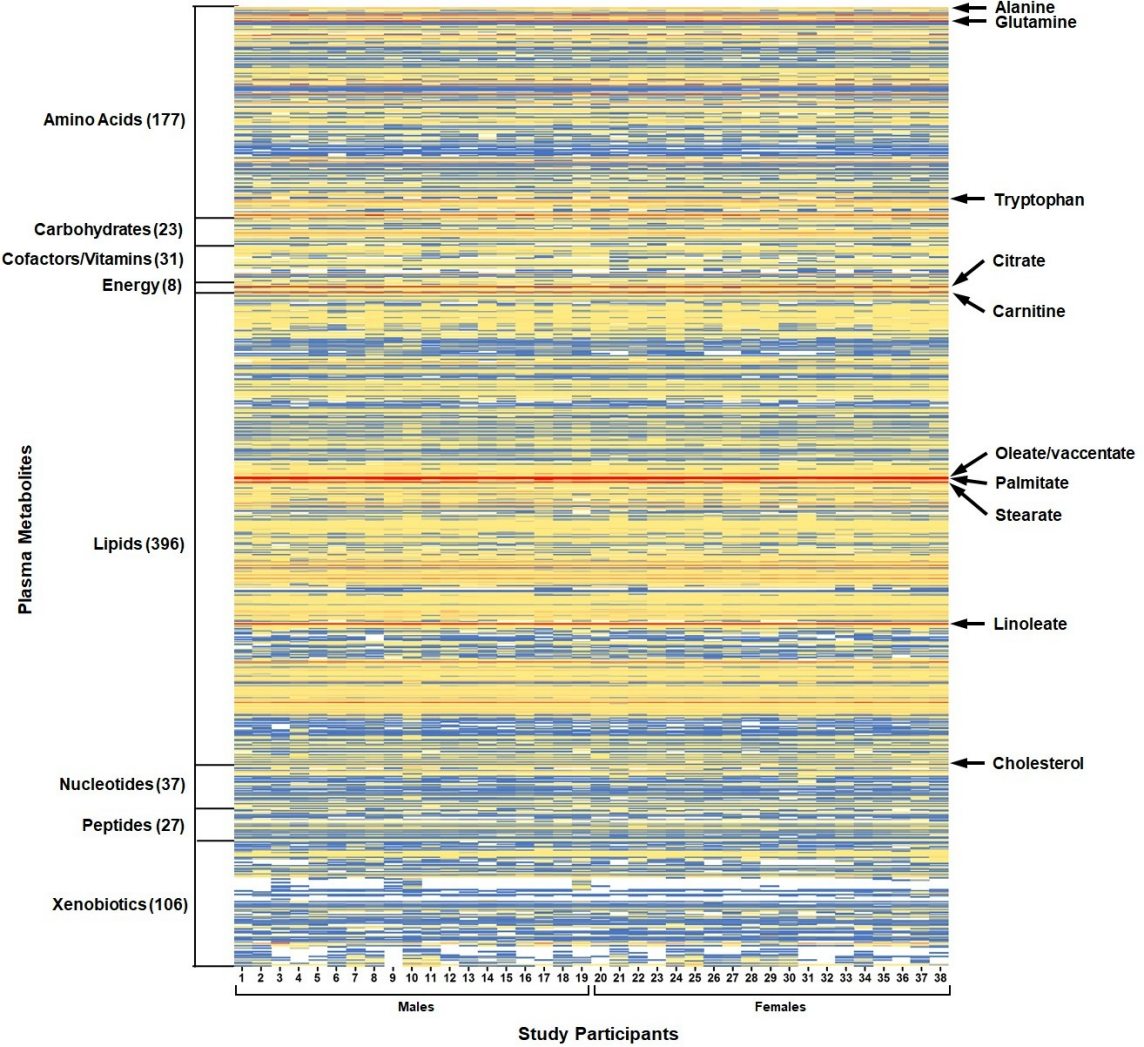
Plasma metabolite profiling of children with aberrant cholesterol. Metabolites identified in plasma (805 total) were normalized across the dataset (median = 1). Metabolites are classified into 8 chemical classes, with the number of metabolites in each chemical class indicated in brackets. Blank (white) cells indicate that metabolite was not detected in the plasma sample.

The plasma metabolite profiles of the 38 children is visualized in Fig 2, which revealed both regions of uniformity and variation. Metabolites with the highest median-scaled relative abundance when compared to all the other metabolites were 5 lipids (carnitine, the long-chain fatty acids oleate/vaccinate, palmitate, and stearate, and the polyunsaturated fatty acid linoleate), 1 amino acid (glutamine) and 1 energy cycle metabolite (citrate) (Fig 2 and S2 Table). The increased abundance of these 7 metabolites was consistent across all children. High relative abundance was also observed for 35 lipids (including cholesterol, Fig 2), 23 amino acids, 3 carbohydrates, 1 cofactor/vitamin, 1 nucleotide, and 1 xenobiotic (S2 Table). The lowest median-scaled relative abundance metabolites when compared to all other metabolites identified in plasma were o-cresol sulfate, xanthurenate, 5-hydroxyindoleacetate, alpha-CEHC sulfate, N6-succinyladenosine, phenylacetylglutamate, and N-(2-furoyl)glycine (S2 Table). Metabolites with low relative abundance also included 26 lipids, 18 amino acids, 11 xenobiotics/phytochemicals, 6 nucleotides, 1 carbohydrate, 1 cofactor/vitamin, and 1 peptide (S2 Table). No significant differences were observed in metabolite levels between males and females.

Several plasma metabolites were identified that correspond to metabolic intermediates or end products for 6 distinct vitamins. Niacin-related metabolites in plasma were quinolinate, 1-methylnicotinamide, nicotinamide, N1-methyl-4-pyridone-3-carboxamide, N1-methyl-2-pyridone-5-carboxamide, trigonelline, and ADP-ribose. Vitamin E metabolites in plasma were alpha-CEHC, alpha-CEHC sulfate, alpha-CEHC glucuronide, alpha-tocopherol, delta-tocopherol, gamma-CEHC, gamma-CEHC glucuronide, and gamma-tocopherol/beta-tocopherol. Riboflavin (vitamin B_2_) metabolites in plasma was flavin adenine dinucleotide (FAD), and retinol for vitamin A. The pyridoxal and pyridoxate were for plasma metabolites for vitamin B_6_, and ascorbate, gulonate, oxalate, and threonate represent plasma metabolites for vitamin C. To assess if self-reported micronutrient intakes align with plasma metabolites of micronutrient status, we compared levels of these metabolites between children whose reported intakes meet the RDA and those who do not. For niacin (vitamin B_3_) and vitamin B_6_, the metabolite relative abundance in children who meet the RDA was higher than children who do not meet the RDA (S1 and S2 Figs). These metabolite patterns with RDA were not observed for riboflavin (vitamin B_2_) (S3 Fig), vitamin A (S3 Fig), and vitamin C (S4 Fig). The median-scaled relative abundance of 6 plasma metabolites for vitamin E were higher in children who meet the RDA compared to those who do not (alpha-CEHC, alpha-CEHC glucuronide, alpha-tocopherol, gamma-CEHC, and gamma-CEHC glucuronide), with a statistically significant difference for alpha-CEHC sulfate (p = 0.0356) (S5 Fig). Notably, the majority of individuals in this cohort did not meet the RDA (36 out of 38 children) for vitamin E. The number of metabolites that comprise total vitamin E intake will require validation with a larger number of participants that consumed varied amounts from the diet.

### Relationships between serum lipids, plasma metabolite profiles, and self-reported micronutrient intakes

**Fig 3:**
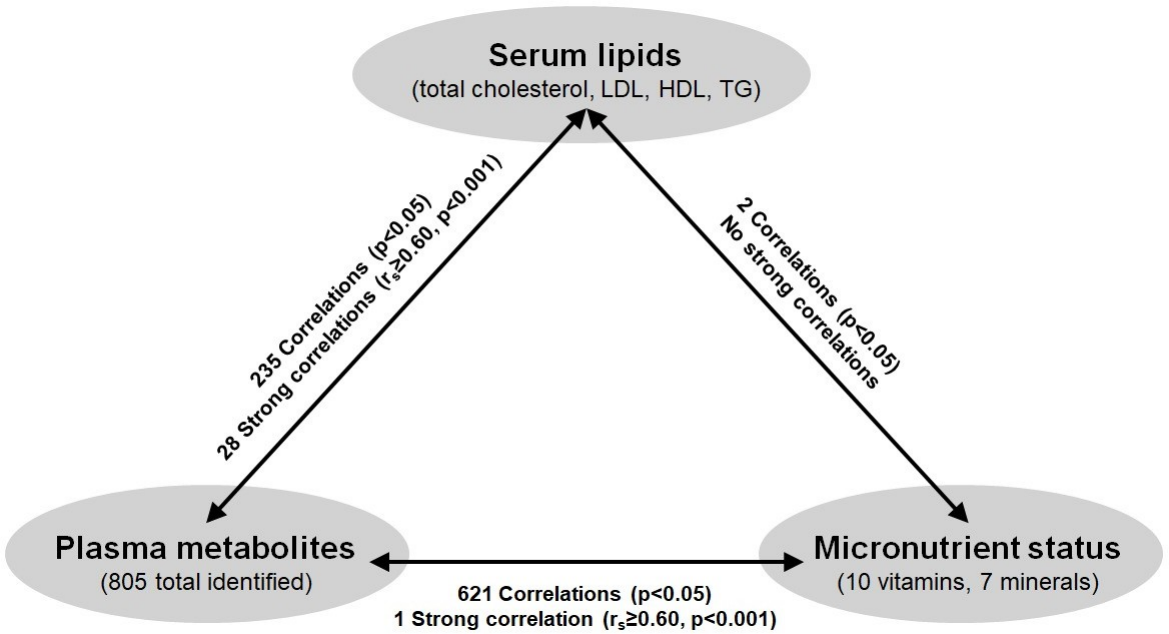
Correlations between serum lipids, plasma metabolite profiles, and micronutrient status. Spearman’s rank-order correlations were performed and significance set at p<0.05 with subsets of strong correlations r_s_≥0.60 and p<0.001. Abbreviations: LDL, low-density lipoprotein cholesterol; HDL, high-density lipoprotein cholesterol; TG, triglycerides.

The 805 plasma metabolites identified from children were next tested for significant correlations to serum lipids. Using a p≤0.05, there were 235 compounds (29% of the plasma metabolome) that met statistical significance (Fig 3). These 157 lipids, 35 amino acids, 13 xenobiotics, 13 peptides, 7 carbohydrates, 7 nucleotides, 2 cofactors/vitamins, and 1 energy cycle metabolite are outlined in S3 Table. The 28 plasma metabolites found to be strongly correlated with serum lipids (r_s_≥0.60; p≤0.0001), and that warranted continued attention as potential biomarkers of CVD risk included, as expected, plasma cholesterol and serum total cholesterol (r_s_ = 0.6654, CI = 0.4312 to 0.8156; p<0.0001). The remaining 27 compounds that correlated with serum TG are listed in Table 2. One plasmalogen [1-(1-enyl-palmitoyl)-2-oleoyl-glycerophosphocholine] was determined to be negatively correlated with serum TG (r_s_ = -0.6806; p<0.0001), and 3 phospholipid metabolites (1-palmitoyl-2-arachidonoyl-glycerophosphoethanolamine, 1-palmitoyl-2-linoleoyl-glycerophosphoethanolamine, and 1-stearoyl-2-linoleoyl-glycerophosphoethanolamine) were positively correlated to serum TG (r_s_ = 0.6281 to 0.7309; p<0.0001). In addition, 68.8% of plasma diacylglycerols identified were strongly correlated with serum TG (r_s_ = 0.6033 to 0.882; p<0.0001). One plasma amino acid, alanine, was also positively correlated with serum TG (r_s_ = 0.6242; p<0.0001).

**Table 2:** Plasma metabolites strongly correlated (r_s_ ≥0.60, p<0.0001) with serum triglycerides in children.

Subpathway	Metabolite	Spearman’s Correlation Coefficient	
		r_s_	95% CI
**Amino Acids**			
Alanine & Aspartate Metabolism	alanine	0.6242	0.372 to 0.7906
**Lipids**			
Plasmalogen	1-(1-enyl-palmitoyl)-2-oleoyl-GPC (P-16:0/18:1)	-0.6806	-0.8247 to -0.4535
Phospholipid Metabolism	1-palmitoyl-2-arachidonoyl-GPE (16:0/20:4)	0.6412	0.3961 to 0.8009
	1-palmitoyl-2-linoleoyl-GPE (16:0/18:2)	0.6281	0.3776 to 0.793
	1-stearoyl-2-linoleoyl-GPE (18:0/18:2)	0.7309	0.5296 to 0.8543
Diacylglycerol	diacylglycerol (12:0/18:1, 14:0/16:1, 16:0/14:1) [2]	0.7975	0.6355 to 0.8922
	diacylglycerol (14:0/18:1, 16:0/16:1) [1]	0.836	0.6997 to 0.9136
	diacylglycerol (14:0/18:1, 16:0/16:1) [2]	0.7833	0.6123 to 0.8842
	diacylglycerol (16:1/18:2 [2], 16:0/18:3 [1])	0.7926	0.6274 to 0.8895
	linoleoyl-linolenoyl-glycerol (18:2/18:3) [1]	0.627	0.376 to 0.7923
	linoleoyl-linolenoyl-glycerol (18:2/18:3) [2]	0.6315	0.3824 to 0.7951
	linoleoyl-linoleoyl-glycerol (18:2/18:2) [1]	0.6033	0.3428 to 0.7776
	oleoyl-linoleoyl-glycerol (18:1/18:2) [1]	0.8187	0.6706 to 0.9041
	oleoyl-linoleoyl-glycerol (18:1/18:2) [2]	0.8092	0.6548 to 0.8988
	oleoyl-oleoyl-glycerol (18:1/18:1) [2]	0.882	0.7793 to 0.9386
	oleoyl-oleoyl-glycerol (18:1/18:1) [1]	0.8605	0.7417 to 0.927
	palmitoleoyl-linoleoyl-glycerol (16:1/18:2) [1]	0.7572	0.5707 to 0.8694
	palmitoleoyl-oleoyl-glycerol (16:1/18:1) [1]	0.6459	0.4029 to 0.8038
	palmitoleoyl-oleoyl-glycerol (16:1/18:1) [2]	0.665	0.4306 to 0.8154
	palmitoyl-linolenoyl-glycerol (16:0/18:3) [2]	0.7017	0.485 to 0.8372
	palmitoyl-linoleoyl-glycerol (16:0/18:2) [1]	0.7086	0.4955 to 0.8413
	palmitoyl-linoleoyl-glycerol (16:0/18:2) [2]	0.8143	0.6633 to 0.9016
	palmitoyl-myristoyl-glycerol (16:0/14:0) [2]	0.7113	0.4995 to 0.8428
	palmitoyl-oleoyl-glycerol (16:0/18:1) [1]	0.8404	0.7071 to 0.916
	palmitoyl-oleoyl-glycerol (16:0/18:1) [2]	0.8603	0.7414 to 0.9269
	palmitoyl-palmitoyl-glycerol (16:0/16:0) [1]	0.6837	0.4581 to 0.8265
	palmitoyl-palmitoyl-glycerol (16:0/16:0) [2]	0.7418	0.5464 to 0.8606

CI, confidence interval; GPC, glycerophosphocholine; GPE, glycerophosphoethanolamine.

Two metabolites were significantly correlated between serum lipids and self-reported micronutrient status, which included negative correlations between beta-carotene with total cholesterol (r_s_ = -0.5096; p = 0.0011) and LDL-cholesterol (r_s_ = -0.386; p = 0.0167) (Fig 3). Additionally, of the 621 significant correlations between plasma metabolites and micronutrient status (p≤0.05) (Fig 3), significant correlations were revealed between vitamins and 50% of the plasma metabolome (403 metabolites) (S4 Table). The other 218 significant correlations were revealed between minerals and represented 27% of the plasma metabolome (p≤0.05) (S5 Table). For vitamins, the strongest correlation identified was between vitamin E and plasma laurylcarnitine (r_s_ = -0.602, CI = -0.7767 to -0.3406; p<0.0001).

## Discussion

The major findings of this study are the plasma metabolites in children that correlate with aberrant cholesterol and inadequate dietary micronutrient intakes. The novel relationships of biological importance to serum lipid regulation and micronutrient status include the diacylglycerol, plasmalogen, phospholipid, and sphingolipid submetabolic pathways. Using a non-targeted metabolomics platform, we determined that 235 plasma metabolites (almost a third of the total plasma metabolome of these children) were significantly correlated with serum lipids, and a subset of 27 metabolites were strongly-correlated with serum TG (r_s_ ≥0.60; p≤0.0001). Given that 27–50% of the plasma metabolites were significantly correlated with micronutrient status, these results generated insights to both clinical and nutritional needs for capturing health status and should be used to improve CVD risk reduction in children.

Since defects in lipid metabolism (resulting from both intrinsic and extrinsic influences) underlie cardiometabolic diseases, elucidation of plasma metabolite signatures associated with aberrant lipids has the potential to enhance standard CVD risk prediction models [4, 5, 33]. Lipids are integral structural components of biological membranes, but they can also mediate cellular processes including oxidation and inflammation, contributing to atherosclerosis [34, 35]. In this cohort, almost 70% of diacylglycerols identified in plasma were strongly correlated with serum TG (r_s_>0.60; p<0.0001). High intracellular diacylglycerol concentrations are implied as a contributing mechanism towards insulin resistance [36], as diacylglycerols are a precursor in the synthesis of TG, as well as a digestive product of dietary TG. Additionally, we observed negative correlations between several plasma plasmalogens and serum TG, including 1-(1-enyl-palmitoyl)-2-oleoyl-GPC, 1-(1-enyl-palmitoyl)-2-palmitoyl-GPC, and 1-(1-enyl-palmitoyl)-2-linoleoyl-GPC (S3 Table). Plasmalogens are subset of membrane glycerophospholipids with roles in cholesterol trafficking, maintenance of cellular membrane integrity, and may act as endogenous plasma antioxidants [37]. Decreased plasmalogen levels have been observed in patients with metabolic syndrome and type II diabetes in conjunction with increased lipid peroxidation and TG levels [38]. There were 3 phospholipid metabolites from this child cohort that were strongly and positively associated with serum TG, and 8 phospholipids that had high relative abundance in plasma when compared to all other lipid metabolites. Additionally, 34 sphingolipids were positively correlated with serum total and LDL-cholesterol, and 12 sphingolipids had high relative abundance in plasma. The role of sphingolipids in the pathogenesis of cardiometabolic diseases is well-documented. Elevated plasma sphingolipids have been observed in adults with type 1 and II diabetes [39], and more recently, altered sphingolipid and glycerophospholipid metabolism were observed during atherosclerotic progression in apolipoprotein E-deficient mice [35].

Aside from lipids, dysregulations in other metabolic pathways are of significance to CVD risk and novel targets of intervention. There was a significant association between two plasma amino acids, alanine and tryptophan (both also had high relative abundance in plasma compared to other metabolites), with serum TG. High alanine and tryptophan levels have previously been documented in hyperlipidemia research studies [40–43]. Moreover, tryptophan is the precursor to serotonin, and excessive release of serotonin from endothelial cells contribute to plaque formation in blood vessels [44]. Not only has tryptophan been detected in atherosclerotic plaques in carotid arteries, it is also known to bind LDL and HDL in the bloodstream, and emerging evidence has shown the kynurenine pathway of tryptophan metabolism can induce inflammatory cytokines (e.g., IFN-γ), which can perpetuate cardiovascular damage [44–46]. Several plasma branched-chain amino acids (BCAAs) (e.g., leucine, isoleucine, butyrylcarnitine, and propionylcarnitine) were also positively correlated with serum TG. Furthermore, the relative abundance of the BCAAs leucine, isoleucine, and valine were also increased compared to all amino acids in the metabolome (S2 and S3 Tables). Elevated levels of BCAAs in blood have previously been associated with metabolomes of obesity in children [6, 15], and with diabetes and coronary artery disease in adults [5, 8, 16]. Moreover, lactate, an end product of glycolysis and a gluconeogenic substrate [47], was determined to be positively associated with serum TG. High blood lactate levels have previously been associated with carotid atherosclerosis in adults [48], and in-hospital mortality in adults with pulmonary embolism [49].

The pilot study limitations included the small sample size, which resulted in inter-individual variability in plasma metabolomes and large confidence intervals for correlation coefficient calculations. Given the preliminary nature of this work, future analyses using multivariate approaches and adjusted p-values is warranted. Additionally, plasma metabolomes from a healthy, normocholesterolemic population of children were not available for the present study. Blood metabolites and nutritional status from children with normal cholesterol levels should be compared to children with abnormal cholesterol levels in order to validate these findings. Furthermore, pubertal status of the children will be an additional variable to consider with these findings at the time of blood collection and dietary intake assessment. Serum lipid levels can fluctuate alongside pubertal changes in children [50] that has not yet been evaluated using the plasma metabolome, and represents an interesting novel direction of this research. Future integration of genetic risk factors for CVD with plasma metabolite profiles could also generate new hypotheses of underlying pathogenesis during childhood.

In addition, all children in this selected Northern Colorado cohort had inadequate intake of one or more micronutrients such that 79 to 100% had inadequate vitamin E intake, calcium, magnesium, vitamin D and/or potassium, and a significant proportion of children (37 to 74%) also had inadequate intakes of vitamin A, thiamine (vitamin B_1_), niacin, folate, vitamin B_6_, and/or zinc (Fig 1B and 1C). These micronutrient inadequacies could have stemmed (in part) from the fact that only 26% of children met recommended daily fruit intake, and only 8% met daily vegetable intake according to recommendations set by the American Heart Association for children aged 9 to 13 years [51] (Table 1). It is not possible to conclude from the current study if these deficiencies are a phenomena specific Northern Colorado children, however, previous reports have indicated that approximately a third of the U.S. population (including children and adults) are at risk for micronutrient deficiencies [52]. Oxidation of LDL-cholesterol, an initiating step in atherosclerosis, is often concurrent with the production of inflammatory cytokines and decreased nitric oxide levels which lead to vasoconstriction and compromised vascular structure [22]. Micronutrients with documented anti-inflammatory, anti-oxidant, and/or immune modulatory effects (e.g., vitamin A, C, D, E, nicotinamide, folate, riboflavin) play significant roles in cardioprotection [22]. The anti-oxidant vitamin E has demonstrated protection via reduced atherosclerotic plaque formation in animal models, and dietary consumption of foods rich in vitamin E has been associated with lower CVD outcomes in clinical studies [53]. Moreover, vitamin B_6_ deficiencies have been linked to multiple metabolic diseases as well as gestational diabetes, and several key enzymes in tryptophan catabolism (to make niacin) are vitamin B_6_-dependent [54]. Conversely, while adequate intake can mitigate disease risk, overt intake can be harmful. In this cohort, 76% children had sodium intake levels above the specified UL. High sodium intake is known to cause hypertension, a high risk factor for coronary atherosclerosis, and recent evidence supports the link between high dietary sodium intake and atherosclerosis in mice and adults with CVD risk factors [55–57]. The correlations between serum lipids and micronutrient status revealed a significant negative correlation between beta-carotene with both total and LDL-cholesterol (Fig 3). In rats fed a high cholesterol diet, dietary supplementation with beta-carotene improved the serum lipid profile and increased fecal excretion of cholesterol compounds, indicating that beta-carotene may act to decrease cholesterol absorption in the intestine, thus facilitating its elimination [58]. While the mechanisms for micronutrient contributions to CVD prevention is well-recognized (e.g., inflammation, anti-oxidation) [22, 59–60], further information on the suite of lipid metabolism impacts is warranted to identify the clinical importance.

Furthermore, the correlations between micronutrient status and plasma metabolites revealed a strong negative correlation between vitamin E and laurylcarnitine, a medium-chain acylcarnitine, in our study. Laurylcarnitine has been linked to fatty acid oxidation disorders, and medium-chain acylcarnitines in general have been reported as markers of polyunsaturated fatty acid (PUFA)-induced peroxisomal fatty acid oxidation [61, 62]. Vitamin E is essential in preventing oxidation of tissue PUFA [63], and along with the correlation finding, the fact that the majority of children in this cohort (95%) were also vitamin E-deficient encapsulates the complexity of plasma metabolome-micronutrient-cholesterol relationships in affecting total CVD risk. Integrating the plasma metabolite profiles of individuals at risk for cardiovascular diseases with traditional risk factors (e.g., cholesterol) could help identify early signs of disease risk and devise more appropriate or sustainable strategies for disease management (e.g., diet and lifestyle interventions).

## Conclusions

This pilot study revealed key relationships between serum lipid levels, plasma metabolite profiles, and micronutrient status in children at risk for CVD. Early identification of CVD risk via plasma metabolite signatures could aid in disease prevention and control strategies for pediatric populations at risk. In addition, integrating micronutrient status with plasma metabolite signatures of CVD risk in children will be critical to fill gaps in our knowledge for how micronutrient-metabolite associations affect cardiovascular health into adulthood.

## Supporting information

S1 Fig Relative abundance of niacin (vitamin B3) pathway plasma metabolites in children meeting or not meeting the RDA for niacin. Plasma metabolites: (a) quinolinate (b) 1-methylnicotinamide (c) nicotinamide (d) N1-methyl-4-pyridone-3-carboxamide (e) N1-methyl-2-pyridone-5-carboxamide (f) trigonelline (g) ADP-ribose.
(TIF)

S2 Fig Relative abundance of vitamin B6 pathway plasma metabolites in in children meeting or not meeting the RDA for vitamin B6. Plasma metabolites: (a) pyridoxal (b) pyridoxate.
(TIF)

S3 Fig Relative abundance of the plasma metabolite (a) riboflavin in children meeting or not meeting the RDA for riboflavin or (b) retinol in children meeting or not meeting the RDA for vitamin A
(TIF)

S4 Fig Relative abundance of vitamin C pathway plasma metabolites in children meeting or not meeting the RDA for vitamin C. Plasma metabolites: (a) threonate (b) oxalate (c) gulonate (d) ascorbate.
(TIF)

S5 Fig Relative abundance of vitamin E pathway plasma metabolites in children meeting or not meeting the RDA for vitamin E. Plasma metabolites: (a) alpha-CEHC (b) alpha-CEHC glucuronide (c) alpha-tocopherol (d) gamma-CEHC (e) gamma-CEHC glucuronide (f) alpha-CEHC sulfate (g) gamma-tocopherol/beta-tocopherol (h) delta-tocopherol.
(TIF)

S1 Table Recommended Daily Allowance and upper level values of selected micronutrients established by the food and nutrition board of the institute of medicine for children 9 to 13 years. (DOCX)

S2 Table Highest and lowest relative abundance metabolites from the plasma metabolome of children with aberrant cholesterol. (DOCX)

S3 Table Plasma metabolites significantly correlated with serum lipids in children. (DOCX)

S4 Table Plasma metabolites significantly correlated with vitamin status in children. (DOCX)

S5 Table Plasma metabolites significantly correlated with mineral status in children. (DOCX)

S1 File Raw and median-scaled relative abundance of metabolites identified in the plasma of children. (XLSX)
