# Supplementary figures and images for: Plasma metabolomics of children with aberrant serum lipids and inadequate micronutrient intake
Source: PLoS One. 2018 Oct 31;13(10):e0205899. doi: 10.1371/journal.pone.0205899 (PMC6209210; doi:10.1371/journal.pone.0205899)

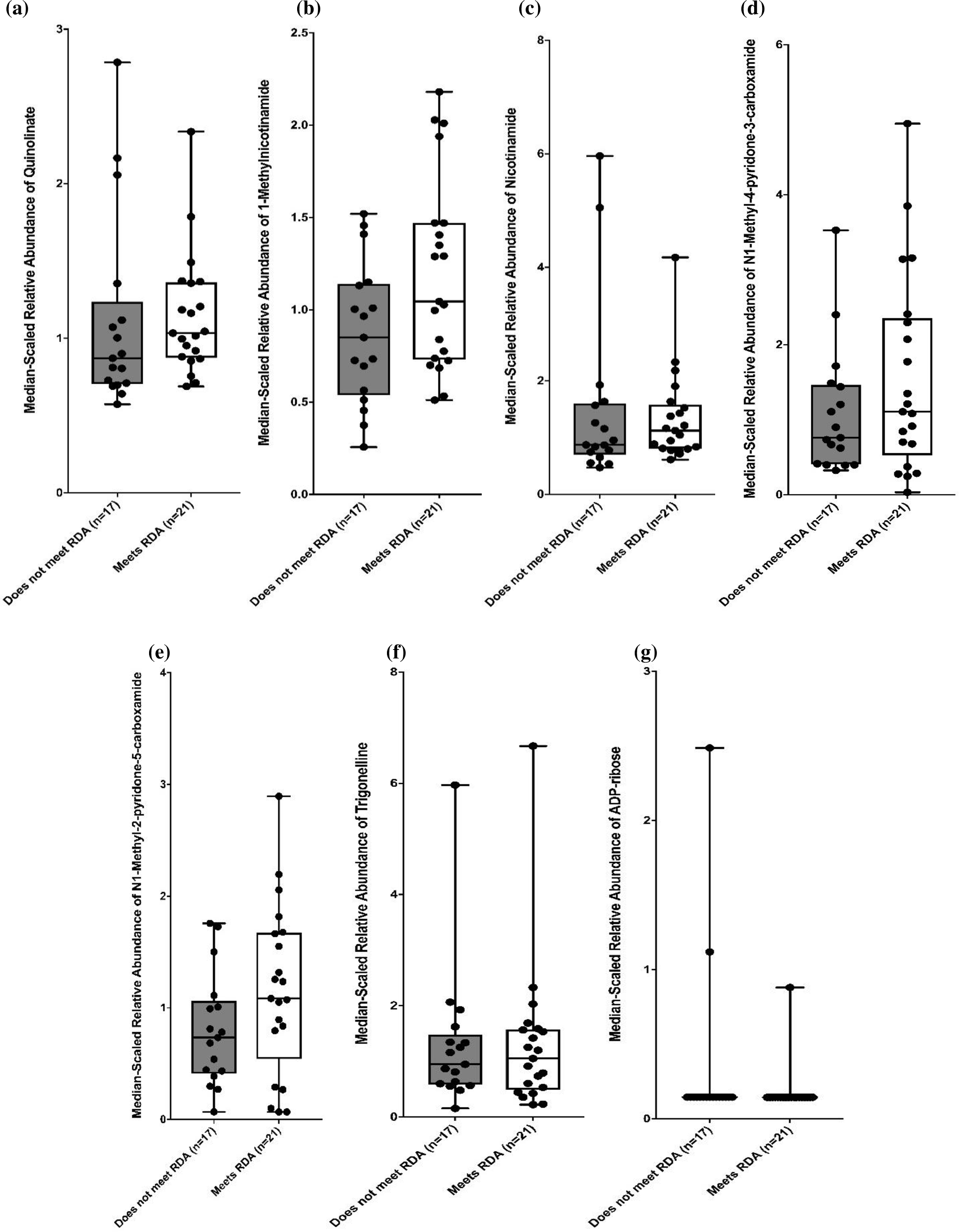

Supplement: S1 Fig — Plasma metabolites: (a) quinolinate (b) 1-methylnicotinamide (c) nicotinamide (d) N1-methyl-4-pyridone-3-carboxamide (e) N1-methyl-2-pyridone-5-carboxamide (f) trigonelline (g) ADP-ribose. (TIF) [file pone.0205899.s001.tif]

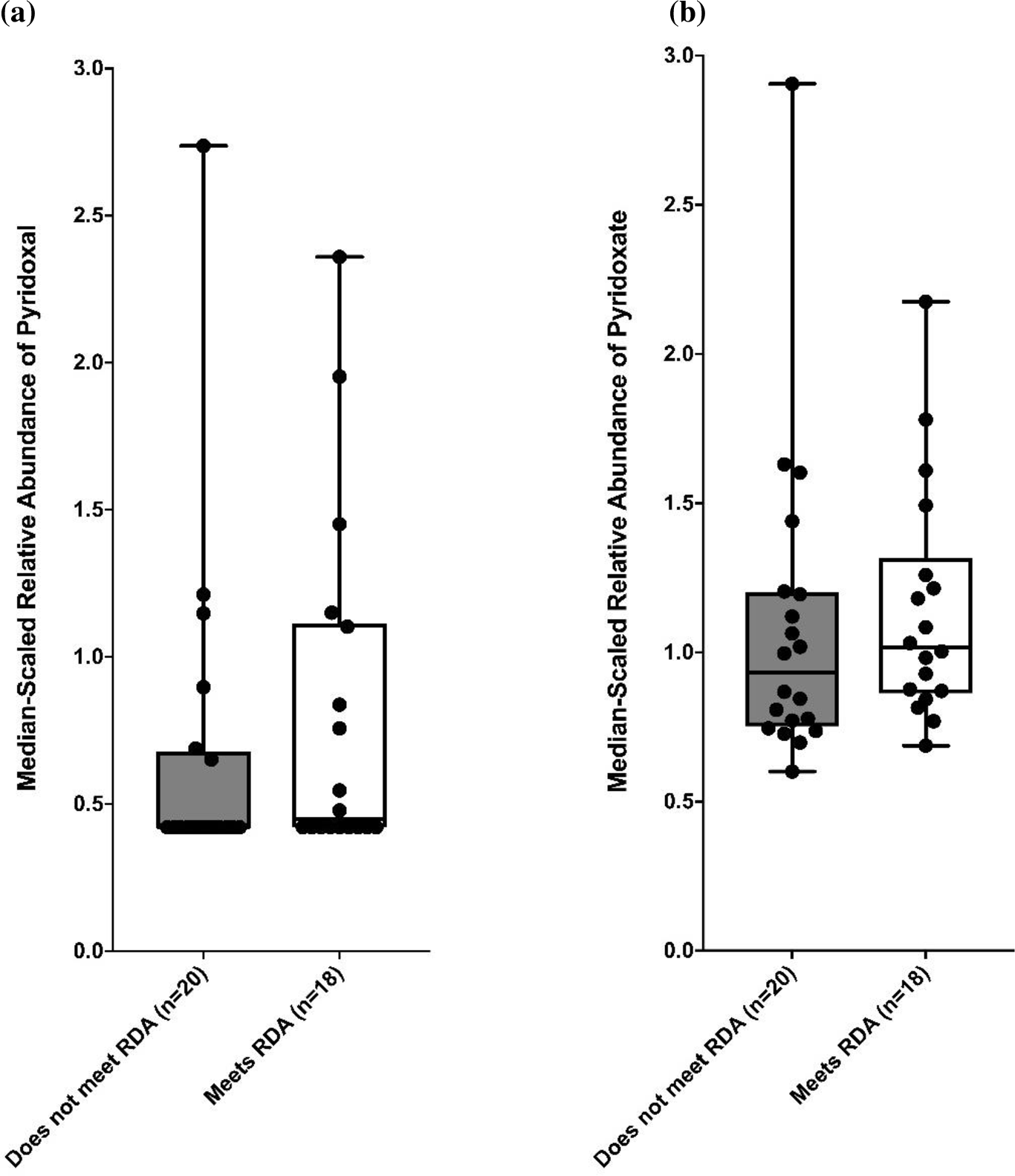

Supplement: S2 Fig — Plasma metabolites: (a) pyridoxal (b) pyridoxate. (TIF) [file pone.0205899.s002.tif]

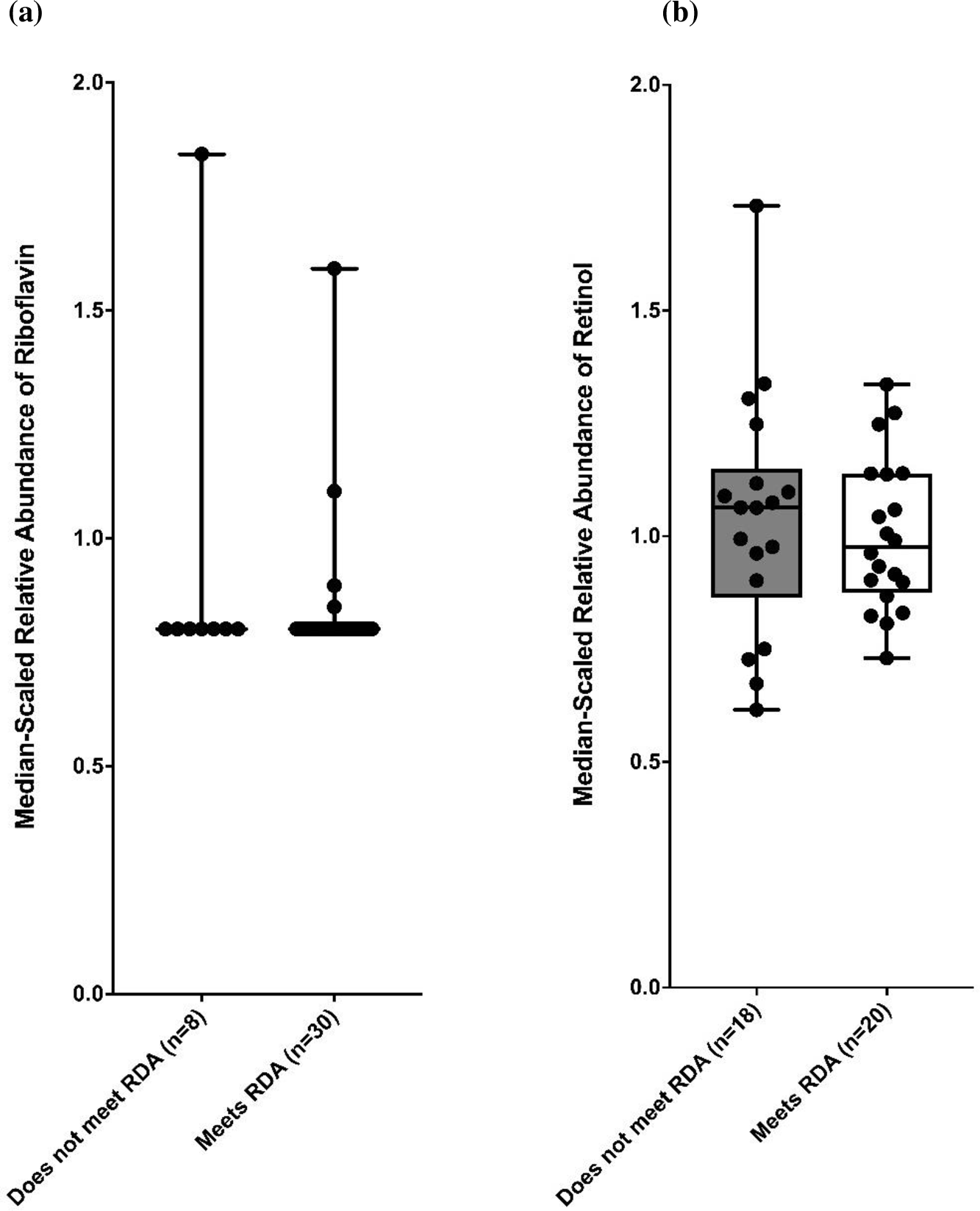

Supplement: S3 Fig — Relative abundance of the plasma metabolite (a) riboflavin in children meeting or not meeting the RDA for riboflavin or (b) retinol in children meeting or not meeting the RDA for vitamin A (TIF) [file pone.0205899.s003.tif]

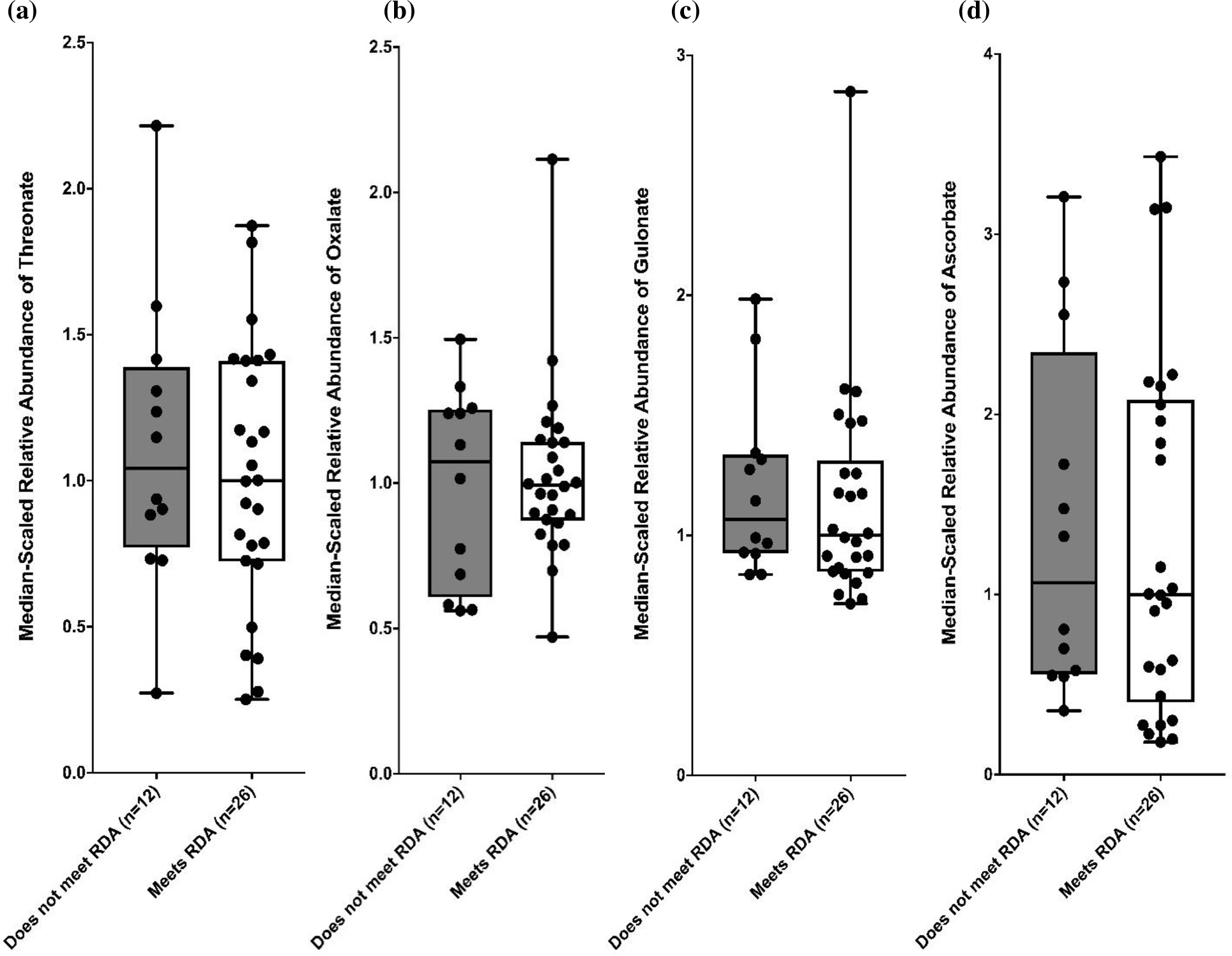

Supplement: S4 Fig — Plasma metabolites: (a) threonate (b) oxalate (c) gulonate (d) ascorbate. (TIF) [file pone.0205899.s004.tif]

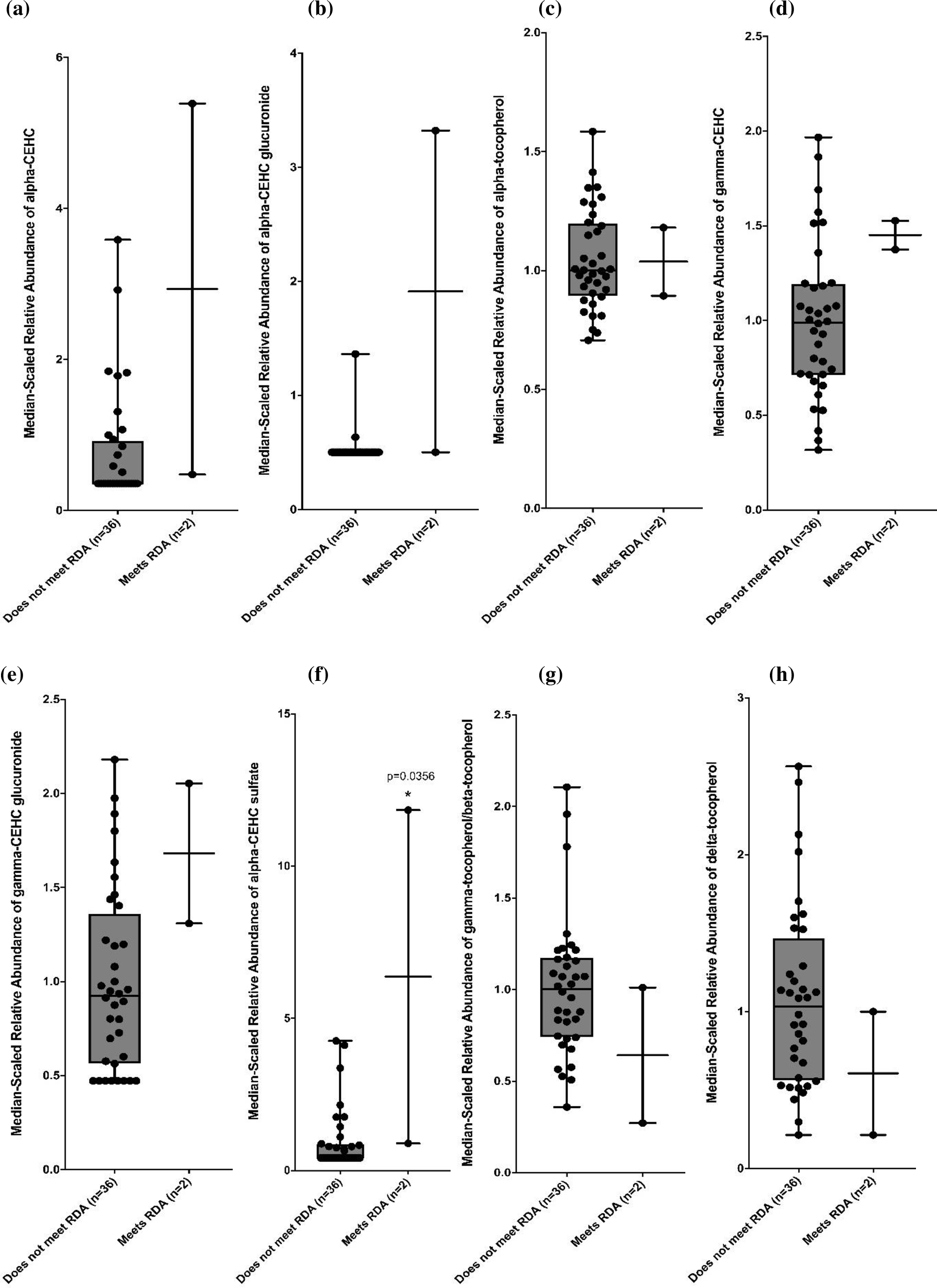

Supplement: S5 Fig — Plasma metabolites: (a) alpha-CEHC (b) alpha-CEHC glucuronide (c) alpha-tocopherol (d) gamma-CEHC (e) gamma-CEHC glucuronide (f) alpha-CEHC sulfate (g) gamma-tocopherol/beta-tocopherol (h) delta-tocopherol. (TIF) [file pone.0205899.s005.tif]
