# Supplementary material for: Plasma metabolomics of children with aberrant serum lipids and inadequate micronutrient intake
Source: PLoS One. 2018 Oct 31;13(10):e0205899. doi: 10.1371/journal.pone.0205899 (PMC6209210; doi:10.1371/journal.pone.0205899)
Supplement: S1 Table — (DOCX) [file pone.0205899.s006.docx]

| **S1 Table Recommended Daily Allowance and Upper Level Values of Selected Micronutrients Established by the**  **Food and Nutrition Board of the Institute of Medicine for Children 9 to 13 years** | | |
| --- | --- | --- |
| **Micronutrient** | **RDA** | **UL** |
| **Vitamins** | | |
| Vitamin A (µg/day) | 600 | 1700 |
| Vitamin C (mg/day) | 45 | 1200 |
| Vitamin D (µg/day) | 15 | 100 |
| Vitamin E (mg/day) | 11 | 600 |
| Thiamine (Vitamin B_1_) (µg/day) | 900 | Not determined |
| Riboflavin (Vitamin B_2_) (µg/day) | 900 | Not determined |
| Niacin (Vitamin B_3_ (mg/day) | 12 | 20 |
| Vitamin B_6_ (mg/day) | 1 | 60 |
| Folate (µg/day) | 300 | 600 |
| Vitamin B_12_ (µg/day) | 1.8 | Not determined |
| **Minerals** | | |
| Zinc (mg/day) | 8 | 23 |
| Calcium (mg/day) | 1300 | 3000 |
| Potassium (mg/day) | 4500 | Not determined |
| Sodium (mg/day) | 1500 | 2200 |
| Iron (mg/day) | 8 | 40 |
| Magnesium (mg/day) | 240 | 350 |
| Selenium (µg/day) | 40 | 280 |

RDA = Recommended Daily Allowance; UL = Upper Level.
