# Supplementary material for: Plasma metabolomics of children with aberrant serum lipids and inadequate micronutrient intake
Source: PLoS One. 2018 Oct 31;13(10):e0205899. doi: 10.1371/journal.pone.0205899 (PMC6209210; doi:10.1371/journal.pone.0205899)
Supplement: S2 Table — (DOCX) [file pone.0205899.s007.docx]

| **S2 Table Highest and Lowest Relative Abundance Metabolites from the Plasma Metabolome of Children with**  **Aberrant Cholesterol** | | | |
| --- | --- | --- | --- |
| **Chemical Class** | **Metabolic Pathway** | **Biochemical Name** | **Median-scaled Relative Abundance^a^** |
| **Metabolites in the Top 1% of Relative Abundance** | | | |
| Lipid | Carnitine Metabolism | carnitine | 709.5373 |
|  | Long Chain Fatty Acid | oleate/vaccinate (18:1) | 2375.518 |
|  |  | palmitate (16:0) | 1754.983 |
|  |  | stearate (18:0) | 1073.025 |
|  | Polyunsaturated Fatty Acid (n3 and n6) | linoleate (18:2n6) | 1524.205 |
| Amino Acid | Glutamate Metabolism | glutamine | 786.5961 |
| Energy | TCA Cycle | citrate | 6473.587 |
| **Metabolites in the Top 10% of Relative Abundance** | | | |
| Lipid | Fatty Acid Metabolism(Acyl Carnitine) | acetylcarnitine (C2) | 220.5038 |
|  | Fatty Acid, Dicarboxylate | adipate | 59.90678 |
|  | Long Chain Fatty Acid | palmitoleate (16:1n7) | 322.0133 |
|  |  | myristate (14:0) | 211.2546 |
|  |  | margarate (17:0) | 43.26604 |
|  | Lysolipid | 1-palmitoyl-GPC (16:0) | 310.2183 |
|  |  | 1-linoleoyl-GPC (18:2) | 167.0542 |
|  |  | 1-stearoyl-GPC (18:0) | 151.0426 |
|  |  | 1-oleoyl-GPC (18:1) | 88.28816 |
|  | Medium Chain Fatty Acid | laurate (12:0) | 42.81485 |
|  | Phospholipid Metabolism | 1-palmitoyl-2-linoleoyl-GPC (16:0/18:2) | 493.3874 |
|  |  | 1-stearoyl-2-linoleoyl-GPC (18:0/18:2) | 372.7946 |
|  |  | 1-palmitoyl-2-arachidonoyl-GPC (16:0/20:4n6) | 290.1504 |
|  |  | 1-palmitoyl-2-oleoyl-GPC (16:0/18:1) | 275.4477 |
|  |  | 1-stearoyl-2-arachidonoyl-GPC (18:0/20:4) | 236.0548 |
|  |  | 1-stearoyl-2-oleoyl-GPC (18:0/18:1) | 117.6947 |
|  |  | choline | 93.21647 |
|  |  | 1,2-dilinoleoyl-GPC (18:2/18:2) | 84.06104 |
|  | Polyunsaturated Fatty Acid (n3 and n6) | linolenate [alpha or gamma; (18:3n3 or 6)] | 166.9865 |
|  |  | arachidonate (20:4n6) | 119.8244 |
|  |  | docosahexaenoate (DHA; 22:6n3) | 50.63304 |
|  |  | dihomo-linolenate (20:3n3 or n6) | 46.09211 |
|  | Sphingolipid Metabolism | sphingomyelin (d18:1/24:1, d18:2/24:0) | 560.0309 |
|  |  | behenoyl sphingomyelin (d18:1/22:0) | 547.829 |
|  |  | palmitoyl sphingomyelin (d18:1/16:0) | 206.6328 |
|  |  | sphingomyelin (d18:2/24:1, d18:1/24:2) | 113.7185 |
|  |  | lignoceroyl sphingomyelin (d18:1/24:0) | 109.4083 |
|  |  | sphingomyelin (d18:1/22:1, d18:2/22:0, d16:1/24:1) | 107.2663 |
|  |  | sphingomyelin (d18:2/16:0, d18:1/16:1) | 78.62721 |
|  |  | sphingomyelin (d18:1/20:0, d16:1/22:0) | 73.01396 |
|  |  | tricosanoyl sphingomyelin (d18:1/23:0) | 69.40192 |
|  |  | sphingomyelin (d18:2/23:0, d18:1/23:1, d17:1/24:1) | 68.08717 |
|  |  | sphingomyelin (d18:1/14:0, d16:1/16:0) | 55.57179 |
|  |  | sphingomyelin (d18:1/21:0, d17:1/22:0, d16:1/23:0) | 43.57749 |
|  | Sterol | cholesterol | 92.25976 |
| Amino Acid | Alanine and Aspartate Metabolism | alanine | 187.5286 |
|  | Creatine Metabolism | creatine | 485.6643 |
|  |  | creatinine | 263.6613 |
|  | Glutamate Metabolism | glutamate | 85.71637 |
|  | Glycine, Serine and Threonine Metabolism | betaine | 378.0245 |
|  |  | threonine | 134.2217 |
|  |  | serine | 55.552 |
|  |  | glycine | 51.95302 |
|  | Leucine, Isoleucine and Valine Metabolism | valine | 435.8351 |
|  |  | leucine | 367.6887 |
|  |  | isoleucine | 356.6649 |
|  | Lysine Metabolism | lysine | 184.3068 |
|  | Methionine, Cysteine, SAM and Taurine Metabolism | methionine | 96.51763 |
|  | Phenylalanine and Tyrosine Metabolism | phenylalanine | 334.6486 |
|  |  | tyrosine | 103.2861 |
|  |  | p-cresol sulfate | 96.54313 |
|  | Tryptophan Metabolism | tryptophan | 192.9928 |
|  | Urea cycle; Arginine and Proline Metabolism | proline | 646.9147 |
|  |  | citrulline | 264.4586 |
|  |  | arginine | 256.999 |
|  |  | urea | 181.6323 |
|  |  | ornithine | 128.9582 |
|  |  | trans-4-hydroxyproline | 92.26344 |
| Carbohydrate | Glycolysis, Gluconeogenesis, and Pyruvate Metabolism | glucose | 328.2539 |
|  |  | lactate | 117.2205 |
|  |  | 1,5-anhydroglucitol (1,5-AG) | 53.4692 |
| Cofactors and Vitamins | Tocopherol Metabolism | alpha-tocopherol | 176.4741 |
| Nucleotide | Purine Metabolism, (Hypo)Xanthine/Inosine containing | urate | 97.12039 |
| Xenobiotics | Food Component/Plant | stachydrine | 79.07454 |
| **Metabolites in the Bottom 1% of Relative Abundance** | | | |
| Amino Acid | Phenylalanine and Tyrosine Metabolism | o-cresol sulfate | 0.012544 |
|  | Tryptophan Metabolism | xanthurenate | 0.010811 |
|  |  | 5-hydroxyindoleacetate | 0.008185 |
| Cofactors and Vitamins | Tocopherol Metabolism | alpha-CEHC sulfate | 0.00609 |
| Nucleotide | Purine Metabolism, Adenine containing | N6-succinyladenosine | 0.011245 |
| Peptide | Acetylated Peptides | phenylacetylglutamate | 0.008395 |
| Xenobiotics | Food Component/Plant | N-(2-furoyl)glycine | 0.004102 |
| **Metabolites in the Bottom 10% of Relative Abundance** | | | |
| Lipid | Diacylglycerol | palmitoyl-myristoyl-glycerol (16:0/14:0) [2] | 0.027772 |
|  | Endocannabinoid | arachidonoyl ethanolamide | 0.055666 |
|  |  | N-stearoyltaurine | 0.053397 |
|  | Fatty Acid Metabolism (Acyl Choline) | oleoylcholine | 0.04123 |
|  |  | stearoylcholine | 0.016368 |
|  | Fatty Acid Metabolism (Acyl Glutamine) | hexanoylglutamine | 0.054738 |
|  | Fatty Acid Metabolism (also BCAA Metabolism) | propionylglycine | 0.03566 |
|  | Fatty Acid Metabolism(Acyl Carnitine) | docosapentaenoylcarnitine (C22:5n3) | 0.047768 |
|  | Fatty Acid Metabolism(Acyl Glycine) | N-linoleoylglycine | 0.040521 |
|  | Fatty Acid, Dicarboxylate | 3-methyladipate | 0.019397 |
|  | Fatty Acid, Monohydroxy | 3-hydroxysebacate | 0.047996 |
|  | Phospholipid Metabolism | glycerophosphoethanolamine | 0.038009 |
|  |  | 1,2-dilinoleoyl-GPE (18:2/18:2) | 0.032828 |
|  |  | glycerophosphoinositol | 0.031836 |
|  |  | arachidonoylcholine | 0.023303 |
|  | Primary Bile Acid Metabolism | glycochenodeoxycholate glucuronide (1) | 0.053728 |
|  |  | taurocholate | 0.048665 |
|  | Secondary Bile Acid Metabolism | 3b-hydroxy-5-cholenoic acid | 0.03028 |
|  |  | 7-ketodeoxycholate | 0.019247 |
|  | Steroid | pregnanediol-3-glucuronide | 0.046504 |
|  |  | 5alpha-androstan-3beta,17alpha-diol disulfate | 0.043483 |
|  |  | corticosterone | 0.03485 |
|  |  | andro steroid monosulfate (1) | 0.030439 |
|  |  | 5alpha-pregnan-3(alpha or beta),20beta-diol disulfate | 0.026682 |
|  |  | 17alpha-hydroxypregnenolone 3-sulfate | 0.020109 |
|  |  | 5alpha-androstan-3beta,17beta-diol monosulfate (2) | 0.016798 |
| Amino Acid | Glutamate Metabolism | N-acetylglutamine | 0.021807 |
|  | Guanidino and Acetamido Metabolism | guanidinosuccinate | 0.020765 |
|  | Histidine Metabolism | hydantoin-5-propionic acid | 0.030707 |
|  | Leucine, Isoleucine and Valine Metabolism | N-acetylleucine | 0.045516 |
|  |  | N-acetylisoleucine | 0.041175 |
|  |  | isobutyrylglycine | 0.02043 |
|  |  | isovalerylglycine | 0.014537 |
|  | Lysine Metabolism | 6-oxopiperidine-2-carboxylate | 0.029841 |
|  | Methionine, Cysteine, SAM and Taurine Metabolism | cysteine sulfinic acid | 0.023415 |
|  | Phenylalanine and Tyrosine Metabolism | dopamine 3-O-sulfate | 0.045264 |
|  |  | homovanillate (HVA) | 0.035147 |
|  |  | tyramine O-sulfate | 0.029937 |
|  |  | vanillactate | 0.028134 |
|  |  | N-acetyltyrosine | 0.023773 |
|  |  | 3-(3-hydroxyphenyl)propionate sulfate | 0.01487 |
|  |  | N-formylphenylalanine | 0.012896 |
|  | Polyamine Metabolism | acisoga | 0.045822 |
|  | Tryptophan Metabolism | N-acetyltryptophan | 0.035613 |
| Xenobiotics | Benzoate Metabolism | 3-(2-hydroxyphenyl)propionate | 0.047 |
|  | Chemical | lanthionine | 0.053913 |
|  |  | 3-hydroxypyridine sulfate | 0.053865 |
|  | Drug | 4-hydroxycoumarin | 0.038508 |
|  |  | ibuprofen | 0.018833 |
|  |  | 4-acetylphenol sulfate | 0.017964 |
|  | Food Component/Plant | eugenol sulfate | 0.049932 |
|  |  | indolin-2-one | 0.036963 |
|  |  | retinal | 0.02004 |
|  |  | acesulfame | 0.015367 |
|  | Xanthine Metabolism | paraxanthine | 0.040558 |
| Nucleotide | Purine Metabolism, Adenine containing | adenosine 3',5'-cyclic monophosphate (cAMP) | 0.050044 |
|  |  | N6-carbamoylthreonyladenosine | 0.036747 |
|  | Pyrimidine Metabolism, Cytidine containing | cytidine | 0.054226 |
|  |  | cytosine | 0.042354 |
|  |  | 2'-O-methylcytidine | 0.024278 |
|  | Pyrimidine Metabolism, Uracil containing | 2'-deoxyuridine | 0.01477 |
| Carbohydrate | Fructose, Mannose and Galactose Metabolism | galactonate | 0.039157 |
| Cofactors and Vitamins | Tocopherol Metabolism | gamma-CEHC glucuronide | 0.020826 |
| Peptide | Dipeptide | valylglutamine | 0.033465 |

^a^ Median-scaled relative abundance of each metabolite for all children. Metabolites with highest/lowest median relative abundance compared to all 805 metabolites identified in plasma were listed as the top/bottom 1% or 10%. Metabolites with median relative abundance = 0 were removed.
