# Supplementary material for: Plasma metabolomics of children with aberrant serum lipids and inadequate micronutrient intake
Source: PLoS One. 2018 Oct 31;13(10):e0205899. doi: 10.1371/journal.pone.0205899 (PMC6209210; doi:10.1371/journal.pone.0205899)
Supplement: S3 Table — (DOCX) [file pone.0205899.s008.docx]

| **S3 Table Plasma Metabolites Significantly Correlated with Serum Lipids in Children** | | | | | |
| --- | --- | --- | --- | --- | --- |
| **Chemical Class/Metabolic Pathway** | **Biochemical Name** | **Spearman’s Correlation Coefficient** | | **P-value** | **Type of Serum Lipid** |
|  |  | **r_s_** | **95% CI** |  |  |
| **Lipids** | | | | | |
| Plasmalogen | 1-(1-enyl-palmitoyl)-2-linoleoyl-GPC (P-16:0/18:2) | -0.3464 | -0.6059 to -0.02021 | 0.0332 | TG |
|  |  | 0.3903 | 0.07091 to 0.6371 | 0.0154 | HDL |
|  | 1-(1-enyl-palmitoyl)-2-oleoyl-GPC (P-16:0/18:1) | -0.6806 | -0.8247 to -0.4535 | <0.0001 | TG |
|  |  | 0.5105 | 0.2188 to 0.7185 | 0.0011 | HDL |
|  | 1-(1-enyl-palmitoyl)-2-oleoyl-GPE (P-16:0/18:1) | 0.3234 | -0.0057 to 0.5892 | 0.0477 | LDL |
|  |  | 0.3639 | 0.04027 to 0.6184 | 0.0247 | Total |
|  | 1-(1-enyl-palmitoyl)-2-palmitoyl-GPC (P-16:0/16:0) | -0.5075 | -0.7165 to -0.2149 | 0.0011 | TG |
|  | 1-(1-enyl-stearoyl)-2-oleoyl-GPE (P-18:0/18:1) | 0.4233 | 0.1102 to 0.66 | 0.0081 | LDL |
|  |  | 0.4415 | 0.1323 to 0.6725 | 0.0055 | Total |
| Fatty Acid, Branched | 15-methylpalmitate | 0.3585 | 0.03403 to 0.6146 | 0.0271 | HDL |
| Steroid | 16a-hydroxy DHEA 3-sulfate | -0.337 | -0.5992 to -0.009657 | 0.0385 | LDL |
|  | 5alpha-androstan-3alpha,17beta-diol disulfate | 0.3723 | 0.04992 to 0.6244 | 0.0214 | TG |
|  | 5alpha-androstan-3alpha,17beta-diol monosulfate (1) | 0.4189 | 0.1049 to 0.657 | 0.0089 | TG |
|  | 5alpha-androstan-3beta,17beta-diol disulfate | 0.4715 | 0.1693 to 0.6927 | 0.0028 | TG |
|  | 5alpha-androstan-3beta,17beta-diol monosulfate (2) | 0.4525 | 0.1457 to 0.6799 | 0.0043 | TG |
|  | andro steroid monosulfate (1) | 0.3451 | 0.01875 to 0.605 | 0.0339 | TG |
|  | androstenediol (3alpha, 17alpha) monsulfate (2) | -0.4057 | -0.6479 to -0.08918 | 0.0115 | LDL |
|  |  | 0.4014 | 0.08408 to 0.6449 | 0.0125 | TG |
|  | androstenediol (3alpha, 17alpha) monsulfate (3) | 0.3434 | 0.01686 to 0.6038 | 0.0348 | TG |
|  | androstenediol (3beta,17beta) disulfate (1) | 0.4401 | 0.1305 to 0.6715 | 0.0057 | TG |
|  | androstenediol (3beta,17beta) disulfate (2) | -0.3224 | -0.5885 to 0.0068 | 0.0484 | LDL |
|  |  | 0.351 | 0.02544 to 0.6092 | 0.0307 | TG |
|  | androstenediol (3beta,17beta) monosulfate (1) | 0.3741 | 0.052 to 0.6256 | 0.0207 | TG |
|  | androstenediol (3beta,17beta) monosulfate (2) | -0.3875 | -0.6351 to -0.06769 | 0.0162 | LDL |
|  |  | 0.4661 | 0.1626 to 0.6891 | 0.0032 | TG |
|  | androsterone sulfate | 0.3385 | 0.01128 to 0.6002 | 0.0377 | TG |
|  | epiandrosterone sulfate | 0.406 | 0.08951 to 0.648 | 0.0114 | TG |
| Lysolipid | 1-arachidonoyl-GPC (20:4n6) | 0.386 | 0.06587 to 0.634 | 0.0167 | LDL |
|  |  | 0.4785 | 0.178 to 0.6973 | 0.0024 | Total |
|  | 1-linoleoyl-GPA (18:2) | 0.3569 | 0.03227 to 0.6135 | 0.0278 | HDL |
|  | 1-stearoyl-GPI (18:0) | 0.3516 | 0.0262 to 0.6097 | 0.0304 | LDL |
|  |  | 0.4437 | 0.1349 to 0.6739 | 0.0053 | Total |
| Monoacylglycerol | 1-myristoylglycerol (14:0) | 0.3592 | 0.03485 to 0.6151 | 0.0268 | TG |
|  | 1-pentadecanoylglycerol (15:0) | -0.3373 | -0.5994 to -0.009987 | 0.0383 | LDL |
| Phospholipid Metabolism | 1-oleoyl-2-linoleoyl-GPE (18:1/18:2) | -0.5111 | -0.7189 to -0.2195 | 0.001 | LDL |
|  |  | -0.3974 | -0.6421 to -0.07932 | 0.0135 | Total |
|  |  | 0.4234 | 0.1103 to 0.6601 | 0.0081 | TG |
|  | 1-palmitoyl-2-arachidonoyl-GPE (16:0/20:4) | -0.3958 | -0.6409 to -0.07744 | 0.0139 | LDL |
|  |  | -0.3646 | -0.6189 to -0.04109 | 0.0244 | HDL |
|  |  | 0.6412 | 0.3961 to 0.8009 | <0.0001 | TG |
|  | 1-palmitoyl-2-arachidonoyl-GPI (16:0/20:4) | 0.4554 | 0.1493 to 0.6819 | 0.0041 | TG |
|  | 1-palmitoyl-2-linoleoyl-GPE (16:0/18:2) | -0.3734 | -0.6252 to -0.05121 | 0.0209 | LDL |
|  |  | 0.6281 | 0.3776 to 0.793 | <0.0001 | TG |
|  | 1-palmitoyl-2-linoleoyl-GPI (16:0/18:2) | -0.3766 | -0.6274 to -0.0549 | 0.0198 | HDL |
|  |  | 0.4058 | 0.08925 to 0.6479 | 0.0115 | TG |
|  | 1-palmitoyl-2-oleoyl-GPC (16:0/18:1) | 0.3447 | 0.01835 to 0.6047 | 0.0341 | TG |
|  | 1-palmitoyl-2-oleoyl-GPE (16:0/18:1) | -0.3829 | -0.6319 to -0.06229 | 0.0177 | LDL |
|  |  | -0.3453 | -0.6051 to -0.01903 | 0.0337 | HDL |
|  |  | 0.5788 | 0.3091 to 0.7623 | 0.0001 | TG |
|  | 1-palmitoyl-2-palmitoleoyl-GPC (16:0/16:1) | 0.5944 | 0.3305 to 0.7721 | <0.0001 | TG |
|  | 1-stearoyl-2-arachidonoyl-GPE (18:0/20:4) | -0.4564 | -0.6825 to -0.1505 | 0.004 | LDL |
|  |  | -0.3853 | -0.6336 to -0.06513 | 0.0169 | HDL |
|  |  | -0.3572 | -0.6137 to -0.0326 | 0.0277 | Total |
|  |  | 0.5594 | 0.2829 to 0.75 | 0.0003 | TG |
|  | 1-stearoyl-2-linoleoyl-GPE (18:0/18:2) | -0.4971 | -0.7097 to -0.2015 | 0.0015 | LDL |
|  |  | 0.7309 | 0.5296 to 0.8543 | <0.0001 | TG |
|  | 1-stearoyl-2-oleoyl-GPE (18:0/18:1) | -0.3777 | -0.6282 to -0.05618 | 0.0194 | HDL |
|  |  | -0.3583 | -0.6144 to -0.0338 | 0.0272 | LDL |
|  | 1-stearoyl-2-oleoyl-GPE (18:0/18:1) | 0.5361 | 0.252 to 0.7351 | 0.0005 | TG |
|  | glycerophosphoinositol | 0.354 | 0.02892 to 0.6114 | 0.0292 | LDL |
|  |  | 0.3574 | 0.03285 to 0.6138 | 0.0276 | Total |
| Fatty Acid, Dicarboxylate | 2-hydroxyadipate | 0.3472 | 0.02115 to 0.6065 | 0.0327 | HDL |
|  | 3-carboxy-4-methyl-5-propyl-2-furanpropanoate (CMPF) | -0.3484 | -0.6074 to -0.02257 | 0.032 | TG |
|  | dodecanedioate | 0.3875 | 0.06769 to 0.6351 | 0.0162 | HDL |
|  | hexadecanedioate | 0.409 | 0.09307 to 0.6501 | 0.0108 | HDL |
|  | tetradecanedioate | 0.5044 | 0.2109 to 0.7145 | 0.0012 | HDL |
|  | octadecanedioate | 0.3292 | 0.0008476 to 0.5935 | 0.0436 | HDL |
| Secondary Bile Acid Metabolism | 3b-hydroxy-5-cholenoic acid | 0.3763 | 0.05459 to 0.6272 | 0.0199 | LDL |
|  | glycocholenate sulfate | 0.3935 | 0.07468 to 0.6393 | 0.0145 | Total |
|  | glycohyocholate | -0.3445 | -0.6045 to -0.01806 | 0.0342 | TG |
|  | glycolithocholate sulfate | 0.324 | -0.004967 to 0.5897 | 0.0472 | LDL |
|  | glycoursodeoxycholate | -0.3549 | -0.612 to -0.02992 | 0.0288 | LDL |
|  | isoursodeoxycholate | -0.3677 | -0.6211 to -0.04465 | 0.0231 | LDL |
|  | tauroursodeoxycholate | -0.3873 | -0.635 to -0.06746 | 0.0163 | TG |
|  | ursodeoxycholate | -0.3374 | -0.5994 to -0.01008 | 0.0383 | LDL |
| Ketone Bodies | 3-hydroxybutyrate (BHBA) | -0.5644 | -0.7532 to -0.2897 | 0.0002 | TG |
|  |  | 0.4451 | 0.1366 to 0.6749 | 0.0051 | HDL |
|  | acetoacetate | -0.5044 | -0.7145 to -0.211 | 0.0012 | TG |
|  |  | 0.4272 | 0.1149 to 0.6627 | 0.0075 | HDL |
| Fatty Acid, Monohydroxy | 3-hydroxyoctanoate | -0.3724 | -0.6245 to -0.05009 | 0.0213 | TG |
|  | 3-hydroxysebacate | -0.4794 | -0.6979 to -0.1791 | 0.0023 | TG |
|  |  | 0.4265 | 0.1141 to 0.6622 | 0.0076 | HDL |
|  | 5-hydroxydecanoate | 0.3773 | 0.0558 to 0.628 | 0.0195 | HDL |
| Long Chain Fatty Acid | arachidate (20:0) | 0.3381 | 0.01084 to 0.5999 | 0.0379 | HDL |
|  | eicosenoate (20:1) | 0.3608 | 0.03667 to 0.6162 | 0.0261 | HDL |
|  | oleate/vaccenate (18:1) | 0.3948 | 0.07619 to 0.6402 | 0.0142 | HDL |
|  | pentadecanoate (15:0) | 0.3344 | 0.006637 to 0.5972 | 0.0402 | HDL |
| Fatty Acid Metabolism (also BCAA Metabolism) | butyrylcarnitine (C4) | 0.4032 | 0.08613 to 0.6461 | 0.0121 | TG |
|  | propionylcarnitine (C3) | 0.4217 | 0.1082 to 0.6589 | 0.0084 | TG |
| Medium Chain Fatty Acid | caproate (6:0) | -0.3387 | -0.6003 to -0.01151 | 0.0375 | LDL |
|  | caprylate (8:0) | -0.3755 | -0.6267 to -0.05369 | 0.0202 | Total |
|  | heptanoate (7:0) | -0.3831 | -0.632 to -0.06254 | 0.0176 | LDL |
|  |  | -0.3661 | -0.62 to -0.04279 | 0.0238 | Total |
|  | laurate (12:0) | 0.3439 | 0.01741 to 0.6041 | 0.0345 | HDL |
| Carnitine Metabolism | carnitine | 0.4238 | 0.1107 to 0.6603 | 0.008 | TG |
| Ceramides | ceramide (d18:1/20:0, d16:1/22:0, d20:1/18:0) | 0.327 | -0.001641 to 0.5919 | 0.0451 | TG |
|  | glycosyl ceramide (d18:2/24:1, d18:1/24:2) | -0.4568 | -0.6828 to -0.151 | 0.0039 | TG |
|  |  | 0.4486 | 0.1409 to 0.6772 | 0.0047 | Total |
|  |  | 0.4658 | 0.1621 to 0.6888 | 0.0032 | LDL |
| Sterol | cholesterol | 0.5966 | 0.3335 to 0.7735 | <0.0001 | LDL |
|  |  | 0.6654 | 0.4312 to 0.8156 | <0.0001 | Total |
|  | 4-cholesten-3-one | 0.5958 | 0.3325 to 0.773 | <0.0001 | TG |
|  | 7-alpha-hydroxy-3-oxo-4-cholestenoate (7-Hoca) | 0.4106 | 0.09498 to 0.6512 | 0.0104 | TG |
|  | campesterol | -0.3905 | -0.6372 to -0.07112 | 0.0154 | TG |
|  |  | 0.4412 | 0.1319 to 0.6722 | 0.0056 | Total |
|  |  | 0.4827 | 0.1834 to 0.7002 | 0.0021 | LDL |
| Fatty Acid Metabolism (Acyl Carnitine) | decanoylcarnitine (C10) | -0.3221 | -0.5883 to 0.007147 | 0.0486 | TG |
|  | acetylcarnitine (C2) | 0.3211 | -0.008224 to 0.5876 | 0.0493 | HDL |
|  | 3-hydroxybutyrylcarnitine (2) | 0.4395 | 0.1298 to 0.6711 | 0.0058 | HDL |
|  | arachidonoylcarnitine (C20:4) | -0.349 | -0.6078 to -0.02318 | 0.0318 | LDL |
|  | dihomo-linolenoylcarnitine (20:3n3 or 6) | -0.3264 | -0.5915 to 0.002274 | 0.0455 | LDL |
|  | docosapentaenoylcarnitine (C22:5n3) | 0.333 | 0.00514 to 0.5963 | 0.041 | TG |
|  | docosatrienoate (22:3n3) | 0.4058 | 0.08926 to 0.6479 | 0.0115 | HDL |
|  | eicosenoylcarnitine (C20:1) | 0.3615 | 0.03755 to 0.6168 | 0.0257 | HDL |
|  | erucoylcarnitine (C22:1) | -0.4506 | -0.6786 to -0.1434 | 0.0045 | TG |
|  | laurylcarnitine (C12) | -0.3769 | -0.6277 to -0.0553 | 0.0197 | TG |
|  | myristoleoylcarnitine (C14:1) | -0.3709 | -0.6234 to -0.04832 | 0.0219 | TG |
|  | palmitoleoylcarnitine (C16:1) | -0.3819 | -0.6312 to -0.06116 | 0.018 | TG |
|  |  | 0.3646 | 0.04109 to 0.6189 | 0.0244 | HDL |
| Fatty Acid Metabolism (Acyl Glutamine) | hexanoylglutamine | -0.4417 | -0.6726 to -0.1324 | 0.0055 | TG |
| Fatty Acid Metabolism (Acyl Glycine) | hexanoylglycine | -0.3259 | -0.5911 to 0.002808 | 0.0458 | Total |
| Polyunsaturated Fatty Acid (n3 and n6) | linoleate (18:2n6) | 0.3381 | 0.01084 to 0.5999 | 0.0379 | HDL |
|  | linolenate [alpha or gamma; (18:3n3 or 6)] | 0.3718 | 0.04944 to 0.6241 | 0.0215 | HDL |
| Diacylglycerol | linoleoyl-arachidonoyl-glycerol (18:2/20:4) [1] | 0.4579 | 0.1524 to 0.6836 | 0.0038 | TG |
|  | linoleoyl-arachidonoyl-glycerol (18:2/20:4) [2] | 0.473 | 0.1712 to 0.6937 | 0.0027 | TG |
|  | linoleoyl-docosahexaenoyl-glycerol (18:2/22:6) [2] | -0.3656 | -0.6197 to -0.04224 | 0.024 | HDL |
|  |  | 0.5086 | 0.2162 to 0.7172 | 0.0011 | TG |
|  | linoleoyl-linolenoyl-glycerol (18:2/18:3) [1] | -0.4987 | -0.7107 to -0.2036 | 0.0014 | LDL |
|  |  | -0.3338 | -0.5968 to -0.005999 | 0.0406 | Total |
|  |  | 0.627 | 0.376 to 0.7923 | <0.0001 | TG |
|  | linoleoyl-linolenoyl-glycerol (18:2/18:3) [2] | -0.3381 | -0.6 to -0.01089 | 0.0379 | LDL |
|  |  | 0.6315 | 0.3824 to 0.7951 | <0.0001 | TG |
|  | linoleoyl-linoleoyl-glycerol (18:2/18:2) [1] | -0.4335 | -0.667 to -0.1225 | 0.0066 | LDL |
|  |  | 0.6033 | 0.3428 to 0.7776 | <0.0001 | TG |
|  | diacylglycerol (12:0/18:1, 14:0/16:1, 16:0/14:1) [1] | 0.5441 | 0.2626 to 0.7403 | 0.0004 | TG |
|  | diacylglycerol (12:0/18:1, 14:0/16:1, 16:0/14:1) [2] | -0.4221 | -0.6592 to -0.1087 | 0.0083 | HDL |
|  |  | 0.7975 | 0.6355 to 0.8922 | <0.0001 | TG |
|  | diacylglycerol (14:0/18:1, 16:0/16:1) [1] | -0.3655 | -0.6196 to -0.0421 | 0.024 | LDL |
|  |  | -0.3625 | -0.6175 to -0.03869 | 0.0253 | HDL |
|  |  | 0.836 | 0.6997 to 0.9136 | <0.0001 | TG |
|  | diacylglycerol (14:0/18:1, 16:0/16:1) [2] | -0.3796 | -0.6296 to -0.05847 | 0.0187 | HDL |
|  |  | 0.7833 | 0.6123 to 0.8842 | <0.0001 | TG |
|  | diacylglycerol (16:1/18:2 [2], 16:0/18:3 [1]) | -0.3642 | -0.6186 to -0.04058 | 0.0246 | HDL |
|  |  | 0.7926 | 0.6274 to 0.8895 | <0.0001 | TG |
|  | oleoyl-arachidonoyl-glycerol (18:1/20:4) [1] | 0.4627 | 0.1583 to 0.6868 | 0.0034 | TG |
|  | oleoyl-arachidonoyl-glycerol (18:1/20:4) [2] | 0.549 | 0.2691 to 0.7434 | 0.0004 | TG |
|  | oleoyl-linolenoyl-glycerol (18:1/18:3) [2] | 0.3854 | 0.06516 to 0.6336 | 0.0169 | TG |
|  | oleoyl-linoleoyl-glycerol (18:1/18:2) [1] | -0.4832 | -0.7005 to -0.1839 | 0.0021 | LDL |
|  |  | 0.8187 | 0.6706 to 0.9041 | <0.0001 | TG |
|  | oleoyl-linoleoyl-glycerol (18:1/18:2) [2] | -0.4149 | -0.6542 to -0.1 | 0.0096 | LDL |
|  |  | -0.3293 | -0.5936 to -0.0009705 | 0.0435 | HDL |
|  |  | 0.8092 | 0.6548 to 0.8988 | <0.0001 | TG |
|  | oleoyl-oleoyl-glycerol (18:1/18:1) [2] | -0.5308 | -0.7317 to -0.2451 | 0.0006 | LDL |
|  |  | -0.3223 | -0.5885 to 0.006879 | 0.0484 | HDL |
|  |  | 0.882 | 0.7793 to 0.9386 | <0.0001 | TG |
|  | oleoyl-oleoyl-glycerol (18:1/18:1)[1] | -0.5132 | -0.7202 to -0.2222 | 0.001 | LDL |
|  |  | 0.8605 | 0.7417 to 0.927 | <0.0001 | TG |
|  | palmitoleoyl-arachidonoyl-glycerol (16:1/20:4) [2] | 0.4812 | 0.1814 to 0.6992 | 0.0022 | TG |
|  | palmitoleoyl-linoleoyl-glycerol (16:1/18:2) [1] | -0.4978 | -0.7102 to -0.2025 | 0.0015 | LDL |
|  |  | 0.7572 | 0.5707 to 0.8694 | <0.0001 | TG |
|  | palmitoleoyl-oleoyl-glycerol (16:1/18:1) [1] | -0.3654 | -0.6195 to -0.04199 | 0.0241 | HDL |
|  |  | -0.3346 | -0.5974 to -0.006902 | 0.04 | LDL |
|  |  | 0.6459 | 0.4029 to 0.8038 | <0.0001 | TG |
|  | palmitoleoyl-oleoyl-glycerol (16:1/18:1) [2] | 0.665 | 0.4306 to 0.8154 | <0.0001 | TG |
|  | palmitoyl-arachidonoyl-glycerol (16:0/20:4) [1] | 0.4214 | 0.1079 to 0.6587 | 0.0084 | TG |
|  | palmitoyl-arachidonoyl-glycerol (16:0/20:4) [2] | -0.3755 | -0.6266 to -0.05363 | 0.0202 | HDL |
|  |  | 0.544 | 0.2624 to 0.7402 | 0.0004 | TG |
|  | palmitoyl-linolenoyl-glycerol (16:0/18:3) [2] | -0.4339 | -0.6673 to -0.123 | 0.0065 | LDL |
|  |  | 0.7017 | 0.485 to 0.8372 | <0.0001 | TG |
|  | palmitoyl-linoleoyl-glycerol (16:0/18:2) [1] | -0.3897 | -0.6367 to -0.07026 | 0.0156 | HDL |
|  |  | -0.3394 | -0.6009 to -0.01238 | 0.0371 | LDL |
|  |  | 0.7086 | 0.4955 to 0.8413 | <0.0001 | TG |
|  | palmitoyl-linoleoyl-glycerol (16:0/18:2) [2] | -0.4037 | -0.6465 to -0.08681 | 0.0119 | HDL |
|  |  | -0.3598 | -0.6155 to -0.03556 | 0.0265 | LDL |
|  |  | 0.8143 | 0.6633 to 0.9016 | <0.0001 | TG |
|  | palmitoyl-myristoyl-glycerol (16:0/14:0) [2] | -0.3512 | -0.6094 to -0.02571 | 0.0306 | HDL |
|  |  | 0.7113 | 0.4995 to 0.8428 | <0.0001 | TG |
|  | palmitoyl-oleoyl-glycerol (16:0/18:1) [1] | -0.4314 | -0.6655 to -0.1199 | 0.0068 | LDL |
|  |  | -0.4108 | -0.6513 to -0.09516 | 0.0104 | HDL |
|  |  | 0.8404 | 0.7071 to 0.916 | <0.0001 | TG |
|  | palmitoyl-oleoyl-glycerol (16:0/18:1) [2] | -0.4503 | -0.6784 to -0.143 | 0.0046 | LDL |
|  |  | -0.39 | -0.6369 to -0.07065 | 0.0155 | HDL |
|  |  | 0.8603 | 0.7414 to 0.9269 | <0.0001 | TG |
|  | palmitoyl-palmitoyl-glycerol (16:0/16:0) [1] | -0.366 | -0.6199 to -0.04266 | 0.0238 | HDL |
|  |  | 0.6837 | 0.4581 to 0.8265 | <0.0001 | TG |
|  | palmitoyl-palmitoyl-glycerol (16:0/16:0) [2] | -0.386 | -0.634 to -0.06587 | 0.0167 | LDL |
|  |  | -0.3508 | -0.6091 to -0.02526 | 0.0308 | HDL |
|  |  | 0.7418 | 0.5464 to 0.8606 | <0.0001 | TG |
| Inositol Metabolism | myo-inositol | -0.3251 | -0.5905 to 0.003706 | 0.0464 | Total |
| Sphingolipid Metabolism | sphinganine-1-phosphate | -0.324 | -0.5897 to 0.00493 | 0.0472 | Total |
|  | sphingomyelin (d17:2/16:0, d18:2/15:0) | 0.3542 | 0.02909 to 0.6115 | 0.0291 | Total |
|  | sphingomyelin (d18:0/18:0, d19:0/17:0) | 0.362 | 0.03807 to 0.6171 | 0.0255 | LDL |
|  |  | 0.3757 | 0.05394 to 0.6268 | 0.0201 | Total |
|  | sphingomyelin (d18:0/20:0, d16:0/22:0) | 0.384 | 0.06356 to 0.6327 | 0.0173 | LDL |
|  |  | 0.3849 | 0.06466 to 0.6333 | 0.017 | Total |
|  | sphingomyelin (d18:1/14:0, d16:1/16:0) | -0.4604 | -0.6852 to -0.1555 | 0.0036 | TG |
|  |  | 0.3642 | 0.04065 to 0.6187 | 0.0246 | Total |
|  |  | 0.4414 | 0.132 to 0.6723 | 0.0055 | LDL |
|  | sphingomyelin (d18:1/15:0, d16:1/17:0) | -0.5406 | -0.738 to -0.2579 | 0.0005 | TG |
|  |  | 0.4014 | 0.08403 to 0.6448 | 0.0125 | LDL |
|  | sphingomyelin (d18:1/17:0, d17:1/18:0, d19:1/16:0) | -0.5499 | -0.744 to -0.2702 | 0.0003 | TG |
|  |  | 0.4287 | 0.1166 to 0.6637 | 0.0072 | LDL |
|  | sphingomyelin (d18:1/19:0, d19:1/18:0) | -0.3429 | -0.6034 to -0.01624 | 0.0351 | TG |
|  |  | 0.3429 | 0.01634 to 0.6034 | 0.035 | LDL |
|  | sphingomyelin (d18:1/20:0, d16:1/22:0) | -0.4442 | -0.6743 to -0.1356 | 0.0052 | TG |
|  | sphingomyelin (d18:1/20:1, d18:2/20:0) | -0.4436 | -0.6738 to -0.1348 | 0.0053 | TG |
|  |  | 0.3747 | 0.05273 to 0.6261 | 0.0205 | LDL |
|  | sphingomyelin (d18:1/20:2, d18:2/20:1, d16:1/22:2) | -0.3932 | -0.6391 to -0.07437 | 0.0146 | TG |
|  | sphingomyelin (d18:1/21:0, d17:1/22:0, d16:1/23:0) | -0.3403 | -0.6015 to -0.01339 | 0.0365 | TG |
|  |  | 0.4115 | 0.09608 to 0.6519 | 0.0103 | Total |
|  |  | 0.4268 | 0.1144 to 0.6624 | 0.0075 | LDL |
|  | sphingomyelin (d18:1/22:1, d18:2/22:0, d16:1/24:1) | -0.4363 | -0.6689 to -0.1259 | 0.0062 | TG |
|  | sphingomyelin (d18:1/22:2, d18:2/22:1, d16:1/24:2) | -0.4113 | -0.6517 to -0.09576 | 0.0103 | TG |
|  |  | 0.3874 | 0.06753 to 0.635 | 0.0163 | LDL |
|  | sphingomyelin (d18:1/24:1, d18:2/24:0) | 0.4237 | 0.1107 to 0.6603 | 0.008 | LDL |
|  |  | 0.4437 | 0.135 to 0.674 | 0.0053 | Total |
|  | sphingomyelin (d18:2/14:0, d18:1/14:1) | 0.3283 | -0.0001508 to 0.5928 | 0.0442 | Total |
|  | sphingomyelin (d18:2/18:1) | -0.3729 | -0.6248 to -0.0506 | 0.0211 | TG |
|  | sphingomyelin (d18:2/21:0, d16:2/23:0) | 0.3357 | 0.008176 to 0.5982 | 0.0393 | LDL |
|  | sphingomyelin (d18:2/23:0, d18:1/23:1, d17:1/24:1) | -0.3834 | -0.6322 to -0.06282 | 0.0175 | TG |
|  |  | 0.5299 | 0.2439 to 0.7311 | 0.0006 | LDL |
|  |  | 0.5543 | 0.2762 to 0.7468 | 0.0003 | Total |
|  | sphingomyelin (d18:2/23:1) | -0.3965 | -0.6414 to -0.07824 | 0.0137 | TG |
|  |  | 0.4555 | 0.1493 to 0.6819 | 0.0041 | Total |
|  |  | 0.5341 | 0.2494 to 0.7338 | 0.0006 | LDL |
|  | sphingomyelin (d18:2/24:1, d18:1/24:2) | -0.419 | -0.6571 to -0.1051 | 0.0088 | TG |
|  |  | 0.4942 | 0.1979 to 0.7078 | 0.0016 | Total |
|  |  | 0.5559 | 0.2782 to 0.7478 | 0.0003 | LDL |
|  | stearoyl sphingomyelin (d18:1/18:0) | -0.4311 | -0.6653 to -0.1196 | 0.0069 | TG |
|  |  | 0.4364 | 0.126 to 0.669 | 0.0062 | Total |
|  |  | 0.5475 | 0.2671 to 0.7425 | 0.0004 | LDL |
|  | behenoyl sphingomyelin (d18:1/22:0) | 0.5585 | 0.2817 to 0.7495 | 0.0003 | LDL |
|  |  | 0.5908 | 0.3255 to 0.7699 | <0.0001 | Total |
|  | glycosyl-N-nervonoyl-sphingosine (d18:1/24:1) | 0.3316 | 0.003496 to 0.5952 | 0.042 | LDL |
|  | glycosyl-N-palmitoyl-sphingosine (d18:1/16:0) | -0.3353 | -0.5979 to -0.007703 | 0.0396 | TG |
|  |  | 0.3597 | 0.03549 to 0.6155 | 0.0265 | Total |
|  |  | 0.4383 | 0.1283 to 0.6703 | 0.0059 | LDL |
|  | glycosyl-N-stearoyl-sphingosine (d18:1/18:0) | 0.4488 | 0.1412 to 0.6774 | 0.0047 | LDL |
|  |  | 0.4641 | 0.1601 to 0.6877 | 0.0033 | Total |
|  | lactosyl-N-nervonoyl-sphingosine (d18:1/24:1) | -0.538 | -0.7364 to -0.2546 | 0.0005 | TG |
|  |  | 0.3695 | 0.04671 to 0.6224 | 0.0224 | Total |
|  |  | 0.4288 | 0.1168 to 0.6637 | 0.0072 | LDL |
|  | lactosyl-N-palmitoyl-sphingosine (d18:1/16:0) | -0.438 | -0.67 to -0.1279 | 0.006 | TG |
|  |  | 0.4676 | 0.1644 to 0.6901 | 0.0031 | Total |
|  |  | 0.49 | 0.1925 to 0.705 | 0.0018 | LDL |
|  | lignoceroyl sphingomyelin (d18:1/24:0) | -0.3417 | -0.6025 to -0.01487 | 0.0358 | TG |
|  |  | 0.4686 | 0.1656 to 0.6907 | 0.003 | Total |
|  |  | 0.4742 | 0.1726 to 0.6945 | 0.0026 | LDL |
|  | tricosanoyl sphingomyelin (d18:1/23:0) | 0.5437 | 0.2621 to 0.74 | 0.0004 | LDL |
|  |  | 0.572 | 0.2999 to 0.758 | 0.0002 | Total |
|  | myristoyl dihydrosphingomyelin (d18:0/14:0) | 0.5069 | 0.2141 to 0.7161 | 0.0012 | LDL |
|  |  | 0.529 | 0.2428 to 0.7306 | 0.0006 | Total |
|  | N-behenoyl-sphingadienine (d18:2/22:0) | -0.3671 | -0.6207 to -0.044 | 0.0234 | LDL |
|  |  | 0.336 | 0.008444 to 0.5984 | 0.0392 | TG |
|  | palmitoyl dihydrosphingomyelin (d18:0/16:0) | -0.3736 | -0.6253 to -0.05149 | 0.0209 | TG |
|  |  | 0.442 | 0.1328 to 0.6728 | 0.0055 | Total |
|  |  | 0.5139 | 0.2231 to 0.7207 | 0.001 | LDL |
|  | palmitoyl sphingomyelin (d18:1/16:0) | -0.5984 | -0.7746 to -0.3359 | <0.0001 | TG |
|  |  | 0.4645 | 0.1606 to 0.688 | 0.0033 | Total |
|  |  | 0.5387 | 0.2554 to 0.7368 | 0.0005 | LDL |
| Lysine Metabolism | 2-aminoadipate | -0.3571 | -0.6136 to -0.0325 | 0.0277 | HDL |
|  |  | 0.4229 | 0.1097 to 0.6597 | 0.0082 | TG |
|  | lysine | 0.4213 | 0.1078 to 0.6586 | 0.0084 | TG |
| Urea cycle; Arginine and Proline Metabolism | 2-oxoarginine | 0.4755 | 0.1743 to 0.6954 | 0.0026 | TG |
|  | argininate | 0.3295 | 0.001181 to 0.5937 | 0.0434 | TG |
|  | homoarginine | -0.3891 | -0.6362 to -0.06949 | 0.0158 | HDL |
|  |  | 0.5623 | 0.2869 to 0.7519 | 0.0002 | TG |
|  | N-methylproline | -0.3477 | -0.6069 to -0.02177 | 0.0324 | HDL |
|  | ornithine | 0.3433 | 0.01673 to 0.6037 | 0.0348 | TG |
|  | proline | 0.4446 | 0.136 to 0.6745 | 0.0052 | TG |
| Polyamine Metabolism | 4-acetamidobutanoate | 0.367 | 0.04389 to 0.6207 | 0.0234 | TG |
| Phenylalanine and Tyrosine Metabolism | 4-hydroxyphenylpyruvate | 0.4087 | 0.09269 to 0.6499 | 0.0108 | TG |
|  | N-acetyltyrosine | -0.3434 | -0.6037 to -0.01684 | 0.0348 | HDL |
|  |  | 0.4633 | 0.1591 to 0.6872 | 0.0034 | TG |
|  | p-cresol sulfate | 0.3276 | -0.0009253 to 0.5923 | 0.0446 | LDL |
|  | tyrosine | -0.5178 | -0.7232 to -0.2281 | 0.0009 | HDL |
|  |  | 0.5359 | 0.2517 to 0.7349 | 0.0005 | TG |
|  | vanillylmandelate (VMA) | -0.3924 | -0.6385 to -0.07339 | 0.0148 | Total |
| Lysine Metabolism | 5-(galactosylhydroxy)-L-lysine | -0.3333 | -0.5965 to -0.005465 | 0.0409 | LDL |
|  |  | 0.3445 | 0.0181 to 0.6045 | 0.0342 | TG |
| Glutathione Metabolism | 5-oxoproline | -0.43 | -0.6646 to -0.1182 | 0.0071 | LDL |
|  |  | -0.4292 | -0.664 to -0.1173 | 0.0072 | Total |
|  | cysteinylglycine | 0.3217 | -0.007513 to 0.588 | 0.0489 | TG |
| Alanine and Aspartate Metabolism | alanine | -0.3305 | -0.5944 to -0.002324 | 0.0427 | HDL |
|  |  | 0.6242 | 0.372 to 0.7906 | <0.0001 | TG |
|  | N-acetylalanine | 0.3715 | 0.04908 to 0.6239 | 0.0216 | TG |
| Methionine, Cysteine, SAM and Taurine Metabolism | cystathionine | 0.4354 | 0.1248 to 0.6683 | 0.0063 | TG |
|  | hypotaurine | -0.3602 | -0.6158 to -0.03599 | 0.0263 | Total |
|  | N-acetylmethionine | -0.4535 | -0.6806 to -0.1469 | 0.0042 | HDL |
| Glutamate Metabolism | glutamate | -0.3495 | -0.6081 to -0.02376 | 0.0315 | HDL |
|  |  | -0.3371 | -0.5992 to -0.009781 | 0.0385 | LDL |
|  |  | 0.4495 | 0.142 to 0.6779 | 0.0046 | TG |
| Tryptophan Metabolism | indoleacetylglutamine | 0.4082 | 0.09209 to 0.6496 | 0.011 | TG |
|  | indolepropionate | 0.3299 | 0.001672 to 0.594 | 0.0431 | TG |
|  | indolepropionylglycine | -0.3453 | -0.6051 to -0.01903 | 0.0337 | HDL |
|  |  | 0.4088 | 0.0928 to 0.65 | 0.0108 | TG |
|  | N-acetyltryptophan | -0.4207 | -0.6582 to -0.1071 | 0.0085 | HDL |
|  |  | 0.5055 | 0.2123 to 0.7152 | 0.0012 | TG |
|  | tryptophan | 0.5469 | 0.2663 to 0.7421 | 0.0004 | TG |
|  | tryptophan betaine | -0.3472 | -0.6065 to -0.02118 | 0.0327 | LDL |
|  | xanthurenate | 0.3775 | 0.05598 to 0.6281 | 0.0195 | TG |
| Leucine, Isoleucine and Valine Metabolism | isoleucine | 0.4131 | 0.09798 to 0.653 | 0.0099 | TG |
|  | leucine | 0.356 | 0.03119 to 0.6128 | 0.0283 | TG |
| Glycine, Serine and Threonine Metabolism | N-acetylglycine | -0.5288 | -0.7304 to -0.2425 | 0.0006 | TG |
|  | N-acetylserine | 0.3621 | 0.03822 to 0.6172 | 0.0255 | TG |
|  | O-acetylhomoserine | 0.347 | 0.02099 to 0.6064 | 0.0328 | HDL |
| **Xenobiotics** | | | | | |
| Xanthine Metabolism | 1,3,7-trimethylurate | 0.327 | -0.001657 to 0.5919 | 0.0451 | Total |
|  | 1,7-dimethylurate | 0.3413 | 0.01449 to 0.6023 | 0.036 | Total |
|  | paraxanthine | 0.3841 | 0.06364 to 0.6327 | 0.0173 | Total |
|  | theophylline | 0.3409 | 0.01405 to 0.602 | 0.0362 | Total |
| Food Component/Plant | 2-keto-3-deoxy-gluconate | -0.3574 | -0.6138 to -0.0328 | 0.0276 | LDL |
|  |  | -0.325 | -0.5904 to 0.003829 | 0.0465 | Total |
|  | acesulfame | -0.3513 | -0.6094 to -0.02583 | 0.0306 | HDL |
|  | beta-cryptoxanthin | -0.3582 | -0.6144 to -0.0337 | 0.0272 | TG |
|  |  | 0.388 | 0.0683 to 0.6355 | 0.0161 | LDL |
|  | methyl glucopyranoside (alpha + beta) | 0.4896 | 0.192 to 0.7047 | 0.0018 | TG |
|  | theanine | 0.4156 | 0.101 to 0.6547 | 0.0095 | Total |
|  |  | 0.4194 | 0.1055 to 0.6573 | 0.0088 | LDL |
| Chemical | N-methylpipecolate | 0.3497 | 0.02401 to 0.6083 | 0.0314 | HDL |
| Benzoate Metabolism | 3-methyl catechol sulfate (2) | 0.3747 | 0.05278 to 0.6261 | 0.0204 | TG |
| Drug | ibuprofen | -0.3448 | -0.6047 to -0.0184 | 0.034 | TG |
|  | S-carboxymethyl-L-cysteine | 0.3263 | -0.002419 to 0.5914 | 0.0456 | TG |
| **Peptide** | | | | | |
| Gamma-glutamyl Amino Acid | gamma-glutamyl-2-aminobutyrate | -0.3442 | -0.6043 to -0.01778 | 0.0343 | TG |
|  | gamma-glutamylalanine | -0.3239 | -0.5897 to 0.005043 | 0.0472 | HDL |
|  |  | 0.4447 | 0.1361 to 0.6746 | 0.0052 | TG |
|  | gamma-glutamyl-alpha-lysine | 0.3494 | 0.0237 to 0.6081 | 0.0315 | TG |
|  | gamma-glutamyl-epsilon-lysine | 0.5016 | 0.2073 to 0.7126 | 0.0013 | TG |
|  | gamma-glutamylglutamine | -0.3275 | -0.5923 to 0.001048 | 0.0447 | LDL |
|  | gamma-glutamylisoleucine | 0.3815 | 0.06065 to 0.6309 | 0.0181 | TG |
|  | gamma-glutamylmethionine | -0.334 | -0.5969 to -0.006204 | 0.0404 | LDL |
|  |  | 0.454 | 0.1475 to 0.6809 | 0.0042 | TG |
|  | gamma-glutamylphenylalanine | -0.3851 | -0.6334 to -0.06483 | 0.017 | HDL |
|  |  | 0.335 | 0.007407 to 0.5977 | 0.0398 | TG |
|  | gamma-glutamyltryptophan | -0.3361 | -0.5985 to -0.008572 | 0.0391 | HDL |
|  |  | 0.3956 | 0.07723 to 0.6408 | 0.0139 | TG |
|  | gamma-glutamyltyrosine | -0.3631 | -0.6178 to -0.03932 | 0.0251 | HDL |
|  |  | 0.416 | 0.1014 to 0.6549 | 0.0094 | TG |
| Dipeptide | prolylglycine | 0.4554 | 0.1493 to 0.6819 | 0.0041 | TG |
| Dipeptide Derivative | N-acetylcarnosine | 0.4105 | 0.09485 to 0.6512 | 0.0105 | TG |
| Acetylated Peptides | phenylacetylglutamate | 0.3296 | 0.00129 to 0.5938 | 0.0433 | LDL |
| **Carbohydrate** | | | | | |
| Glycolysis, Gluconeogenesis, and Pyruvate Metabolism | 1,5-anhydroglucitol (1,5-AG) | -0.4001 | -0.6439 to -0.08248 | 0.0128 | LDL |
|  |  | -0.3504 | -0.6088 to -0.02485 | 0.031 | Total |
|  | lactate | -0.4352 | -0.6682 to -0.1246 | 0.0063 | LDL |
|  |  | 0.5688 | 0.2956 to 0.756 | 0.0002 | TG |
|  | pyruvate | 0.4291 | 0.1172 to 0.664 | 0.0072 | TG |
| Pentose Metabolism | arabitol/xylitol | 0.4197 | 0.1058 to 0.6575 | 0.0087 | TG |
|  | ribitol | -0.3615 | -0.6168 to -0.03755 | 0.0257 | HDL |
|  |  | 0.5558 | 0.2781 to 0.7477 | 0.0003 | TG |
| Advanced Glycation End-product | N6-carboxymethyllysine | -0.4448 | -0.6747 to -0.1362 | 0.0051 | HDL |
|  |  | 0.4781 | 0.1775 to 0.6971 | 0.0024 | TG |
| Aminosugar Metabolism | N-acetylglucosamine/N-acetylgalactosamine | 0.3695 | 0.04667 to 0.6224 | 0.0224 | TG |
| **Nucleotide** | | | | | |
| Pyrimidine Metabolism, Thymine containing | 3-aminoisobutyrate | 0.3586 | 0.03416 to 0.6146 | 0.0271 | HDL |
| Pyrimidine Metabolism, Cytidine containing | 3-methylcytidine | 0.3264 | -0.002254 to 0.5915 | 0.0455 | TG |
| Pyrimidine Metabolism, Uracil containing | 3-ureidopropionate | 0.3274 | -0.001171 to 0.5922 | 0.0448 | LDL |
| Pyrimidine Metabolism, Orotate containing | dihydroorotate | 0.3386 | 0.01137 to 0.6003 | 0.0376 | TG |
| Purine Metabolism, Guanine containing | 7-methylguanine | 0.4349 | 0.1242 to 0.668 | 0.0064 | TG |
| Purine Metabolism, Adenine containing | N1-methyladenosine | -0.3447 | -0.6047 to -0.01828 | 0.0341 | HDL |
|  |  | 0.454 | 0.1475 to 0.6809 | 0.0042 | TG |
| Purine Metabolism, (Hypo)Xanthine/Inosine containing | urate | 0.4336 | 0.1226 to 0.6671 | 0.0065 | TG |
| **Cofactors and Vitamins** | | | | | |
| Hemoglobin and Porphyrin Metabolism | bilirubin (E,E) | 0.4452 | 0.1367 to 0.6749 | 0.0051 | HDL |
| Vitamin A Metabolism | retinol (Vitamin A) | 0.347 | 0.02096 to 0.6064 | 0.0328 | TG |
| **Energy** | | | | | |
| TCA Cycle | succinylcarnitine (C4-DC) | -0.4795 | -0.698 to -0.1793 | 0.0023 | HDL |
|  |  | 0.5138 | 0.223 to 0.7207 | 0.001 | TG |

CI, confidence interval; GPA, glycerophosphatidic acid; GPC, glycerophosphocholine; GPE, glycerophosphoethanolamine; GPI, glycerophosphoinositol; HDL, high-density lipoprotein; LDL, low-density lipoprotein; TG, triglycerides.
