# Supplementary material for: Plasma metabolomics of children with aberrant serum lipids and inadequate micronutrient intake
Source: PLoS One. 2018 Oct 31;13(10):e0205899. doi: 10.1371/journal.pone.0205899 (PMC6209210; doi:10.1371/journal.pone.0205899)
Supplement: S4 Table — (DOCX) [file pone.0205899.s009.docx]

| **S4 Table Plasma Metabolites Significantly Correlated with Vitamin Status in Children** | | | | |
| --- | --- | --- | --- | --- |
| **Micronutrient** | **Biochemical Name** | **Spearman’s Correlation Coefficient** | | **P-value** |
|  |  | **r_s_** | **95% CI** |  |
| Alpha-Tocopherol | 2-aminobutyrate | -0.342 | -0.6025 to -0.01495 | 0.0357 |
|  | 2-hydroxybutyrate/2-hydroxyisobutyrate | -0.377 | -0.6275 to -0.05511 | 0.0197 |
|  | 2-hydroxydecanoate | 0.3308 | 0.002616 to 0.5946 | 0.0425 |
|  | 3-hydroxypyridine sulfate | 0.3244 | -0.004494 to 0.59 | 0.0469 |
|  | 3-methyl catechol sulfate (1) | 0.3629 | 0.03907 to 0.6177 | 0.0252 |
|  | adrenate (22:4n6) | -0.372 | -0.6244 to -0.05003 | 0.0213 |
|  | aspartate | 0.3325 | 0.004583 to 0.5959 | 0.0414 |
|  | ceramide (d18:2/24:1, d18:1/24:2) | 0.4991 | 0.2041 to 0.711 | 0.0014 |
|  | C-glycosyltryptophan | -0.331 | -0.5951 to -0.003353 | 0.0421 |
|  | glycodeoxycholate sulfate | -0.339 | -0.6006 to -0.01192 | 0.0373 |
|  | guanosine | -0.335 | -0.5975 to -0.007032 | 0.04 |
|  | N-acetylcarnosine | 0.3522 | 0.02689 to 0.6101 | 0.0301 |
|  | N-methylpipecolate | -0.333 | -0.5961 to -0.004829 | 0.0412 |
|  | N-nervonoyl-hexadecasphingosine (d16:1/24:1) | 0.3533 | 0.02814 to 0.6109 | 0.0296 |
|  | o-cresol sulfate | 0.3403 | 0.01339 to 0.6015 | 0.0365 |
|  | oleoyl-linolenoyl-glycerol (18:1/18:3) [2] | 0.3405 | 0.01359 to 0.6017 | 0.0364 |
|  | orotate | -0.433 | -0.6663 to -0.1213 | 0.0067 |
|  | pyridoxal | 0.4719 | 0.1697 to 0.6929 | 0.0028 |
|  | pyroglutamine | 0.357 | 0.03239 to 0.6135 | 0.0278 |
| Beta-carotene | 1-(1-enyl-palmitoyl)-2-oleoyl-GPC (P-16:0/18:1) | -0.326 | -0.591 to 0.003026 | 0.046 |
|  | 12-HETE | 0.3995 | 0.08178 to 0.6435 | 0.013 |
|  | 1-arachidonoyl-GPC (20:4n6) | -0.35 | -0.6087 to -0.02464 | 0.0311 |
|  | 2-aminoadipate | 0.3821 | 0.06134 to 0.6313 | 0.0179 |
|  | 2-aminooctanoate | -0.358 | -0.6142 to -0.03339 | 0.0274 |
|  | 3beta,7beta-dihydroxy-5-cholestenoate | 0.373 | 0.05079 to 0.6249 | 0.0211 |
|  | 3-methylglutarate/2-methylglutarate | 0.3409 | 0.01405 to 0.602 | 0.0362 |
|  | 5alpha-androstan-3alpha,17beta-diol monosulfate (1) | 0.4022 | 0.08499 to 0.6454 | 0.0123 |
|  | 5alpha-androstan-3beta,17beta-diol monosulfate (2) | 0.3891 | 0.06952 to 0.6362 | 0.0158 |
|  | 5alpha-pregnan-3(alpha or beta),20beta-diol disulfate | 0.3568 | 0.03207 to 0.6133 | 0.0279 |
|  | 5alpha-pregnan-3beta,20beta-diol monosulfate (1) | 0.4256 | 0.113 to 0.6616 | 0.0077 |
|  | androstenediol (3alpha, 17alpha) monsulfate (3) | 0.3717 | 0.04927 to 0.624 | 0.0216 |
|  | androstenediol (3beta,17beta) monosulfate (2) | 0.3445 | 0.01807 to 0.6045 | 0.0342 |
|  | androsterone sulfate | 0.3376 | 0.01025 to 0.5995 | 0.0382 |
|  | carboxyibuprofen | 0.3277 | -0.0008703 to 0.5924 | 0.0446 |
|  | C-glycosyltryptophan | -0.43 | -0.6647 to -0.1184 | 0.007 |
|  | gamma-glutamylleucine | 0.3203 | -0.009134 to 0.587 | 0.05 |
|  | glycocholate | 0.375 | 0.05307 to 0.6263 | 0.0204 |
|  | imidazole lactate | -0.377 | -0.6274 to -0.05485 | 0.0198 |
|  | lysine | 0.329 | 0.000651 to 0.5934 | 0.0437 |
|  | methionine | 0.3826 | 0.06199 to 0.6317 | 0.0177 |
|  | N6-succinyladenosine | -0.345 | -0.6046 to -0.01825 | 0.0341 |
|  | N-acetylglycine | -0.398 | -0.6421 to -0.07945 | 0.0135 |
|  | perfluorooctanesulfonic acid (PFOS) | 0.4172 | 0.1029 to 0.6558 | 0.0092 |
|  | phenylacetylglutamine | -0.362 | -0.6168 to -0.03766 | 0.0257 |
|  | picolinate | 0.4271 | 0.1147 to 0.6626 | 0.0075 |
|  | pimeloylcarnitine/3-methyladipoylcarnitine (C7-DC) | -0.369 | -0.6222 to -0.04642 | 0.0225 |
|  | pregnenolone sulfate | 0.3772 | 0.05562 to 0.6278 | 0.0196 |
|  | quinolinate | 0.3207 | -0.008646 to 0.5873 | 0.0496 |
|  | sphingomyelin (d18:1/20:1, d18:2/20:0) | -0.351 | -0.6095 to -0.02589 | 0.0305 |
|  | sphingomyelin (d18:2/18:1) | -0.323 | -0.5891 to 0.005961 | 0.0478 |
|  | tauro-beta-muricholate | -0.348 | -0.6067 to -0.02157 | 0.0325 |
|  | tyrosine | 0.4415 | 0.1322 to 0.6724 | 0.0055 |
|  | uridine | 0.4144 | 0.09947 to 0.6538 | 0.0097 |
| Vitamin A | 1-(1-enyl-palmitoyl)-2-oleoyl-GPC (P-16:0/18:1) | -0.321 | -0.5875 to 0.008402 | 0.0495 |
|  | 17alpha-hydroxypregnenolone 3-sulfate | 0.3288 | 0.0003802 to 0.5932 | 0.0439 |
|  | 21-hydroxypregnenolone disulfate | 0.3743 | 0.05231 to 0.6258 | 0.0206 |
|  | 3beta,7beta-dihydroxy-5-cholestenoate | 0.4098 | 0.09398 to 0.6507 | 0.0106 |
|  | 3-hydroxylaurate | 0.3509 | 0.02539 to 0.6092 | 0.0308 |
|  | 3-methylcytidine | 0.4689 | 0.166 to 0.6909 | 0.003 |
|  | 3-methylglutarate/2-methylglutarate | 0.4537 | 0.1471 to 0.6807 | 0.0042 |
|  | 5alpha-androstan-3alpha,17beta-diol disulfate | 0.4319 | 0.1206 to 0.6659 | 0.0068 |
|  | 5alpha-androstan-3alpha,17beta-diol monosulfate (1) | 0.5119 | 0.2205 to 0.7194 | 0.001 |
|  | 5alpha-androstan-3beta,17beta-diol disulfate | 0.4258 | 0.1132 to 0.6617 | 0.0077 |
|  | 5alpha-androstan-3beta,17beta-diol monosulfate (2) | 0.5108 | 0.2191 to 0.7187 | 0.0011 |
|  | 5alpha-pregnan-3(alpha or beta),20beta-diol disulfate | 0.4478 | 0.1399 to 0.6767 | 0.0048 |
|  | 5alpha-pregnan-3beta,20alpha-diol disulfate | 0.4054 | 0.0888 to 0.6476 | 0.0116 |
|  | 5alpha-pregnan-3beta,20alpha-diol monosulfate (2) | 0.4142 | 0.09921 to 0.6537 | 0.0097 |
|  | 5alpha-pregnan-3beta,20beta-diol monosulfate (1) | 0.5362 | 0.2521 to 0.7352 | 0.0005 |
|  | androstenediol (3alpha, 17alpha) monsulfate (2) | 0.3872 | 0.06726 to 0.6349 | 0.0163 |
|  | androstenediol (3alpha, 17alpha) monsulfate (3) | 0.4724 | 0.1704 to 0.6933 | 0.0027 |
|  | androstenediol (3beta,17beta) disulfate (2) | 0.3496 | 0.0239 to 0.6082 | 0.0314 |
|  | androstenediol (3beta,17beta) monosulfate (2) | 0.433 | 0.1219 to 0.6666 | 0.0066 |
|  | androsterone sulfate | 0.4544 | 0.1481 to 0.6812 | 0.0042 |
|  | epiandrosterone sulfate | 0.4247 | 0.1118 to 0.6609 | 0.0079 |
|  | gamma-glutamylleucine | 0.3614 | 0.03741 to 0.6167 | 0.0258 |
|  | glutamate | 0.3514 | 0.02589 to 0.6095 | 0.0305 |
|  | lysine | 0.3632 | 0.03942 to 0.6179 | 0.025 |
|  | methionine | 0.4321 | 0.1208 to 0.666 | 0.0067 |
|  | N6-succinyladenosine | -0.348 | -0.6071 to -0.02213 | 0.0322 |
|  | N-acetylglycine | -0.476 | -0.6955 to -0.1745 | 0.0025 |
|  | N-acetylisoleucine | 0.3614 | 0.03743 to 0.6167 | 0.0258 |
|  | perfluorooctanesulfonic acid (PFOS) | 0.494 | 0.1977 to 0.7077 | 0.0016 |
|  | phenylalanine | 0.3349 | 0.007292 to 0.5976 | 0.0398 |
|  | picolinate | 0.4669 | 0.1635 to 0.6896 | 0.0031 |
|  | pregnenolone sulfate | 0.3568 | 0.03214 to 0.6134 | 0.0279 |
|  | pyridoxal | 0.3511 | 0.02558 to 0.6093 | 0.0307 |
|  | quinolinate | 0.3352 | 0.007538 to 0.5978 | 0.0397 |
|  | sphingomyelin (d18:1/20:1, d18:2/20:0) | -0.347 | -0.6066 to -0.02141 | 0.0326 |
|  | sphingomyelin (d18:2/18:1) | -0.337 | -0.5987 to -0.009018 | 0.0389 |
|  | tauro-beta-muricholate | -0.358 | -0.6145 to -0.03395 | 0.0271 |
|  | tyrosine | 0.3601 | 0.0359 to 0.6157 | 0.0264 |
| Vitamin B1 | 13-HODE + 9-HODE | -0.344 | -0.6041 to -0.01743 | 0.0345 |
|  | 1-lignoceroyl-GPC (24:0) | -0.352 | -0.6102 to -0.02704 | 0.03 |
|  | 2-methoxyresorcinol sulfate | 0.401 | 0.08351 to 0.6445 | 0.0126 |
|  | 3-methylcytidine | 0.4198 | 0.106 to 0.6576 | 0.0087 |
|  | 3-methylglutarate/2-methylglutarate | 0.4026 | 0.08551 to 0.6457 | 0.0122 |
|  | 5alpha-androstan-3alpha,17beta-diol disulfate | 0.5206 | 0.2318 to 0.7251 | 0.0008 |
|  | 5alpha-androstan-3alpha,17beta-diol monosulfate (1) | 0.346 | 0.01975 to 0.6056 | 0.0334 |
|  | 5alpha-androstan-3beta,17alpha-diol disulfate | 0.3508 | 0.02524 to 0.6091 | 0.0308 |
|  | 5alpha-androstan-3beta,17beta-diol disulfate | 0.4585 | 0.1531 to 0.6839 | 0.0038 |
|  | 5alpha-androstan-3beta,17beta-diol monosulfate (2) | 0.3815 | 0.06061 to 0.6309 | 0.0181 |
|  | 5alpha-pregnan-3beta,20alpha-diol disulfate | 0.3973 | 0.07922 to 0.642 | 0.0135 |
|  | 5alpha-pregnan-3beta,20alpha-diol monosulfate (2) | 0.3312 | 0.003107 to 0.5949 | 0.0422 |
|  | 5-hydroxyhexanoate | 0.3752 | 0.05333 to 0.6265 | 0.0203 |
|  | 7-methylguanine | 0.3336 | 0.005813 to 0.5967 | 0.0407 |
|  | adenosine 3',5'-cyclic monophosphate (cAMP) | 0.3421 | 0.01534 to 0.6028 | 0.0355 |
|  | adipoylcarnitine (C6-DC) | 0.3717 | 0.04927 to 0.624 | 0.0216 |
|  | alpha-CEHC glucuronide | 0.3217 | -0.007549 to 0.588 | 0.0489 |
|  | androstenediol (3alpha, 17alpha) monsulfate (3) | 0.3542 | 0.02914 to 0.6115 | 0.0291 |
|  | androsterone sulfate | 0.3783 | 0.05689 to 0.6286 | 0.0192 |
|  | arabitol/xylitol | 0.3995 | 0.08178 to 0.6435 | 0.013 |
|  | aspartate | 0.3634 | 0.03967 to 0.6181 | 0.0249 |
|  | beta-hydroxyisovalerate | 0.3345 | 0.006799 to 0.5973 | 0.0401 |
|  | catechol sulfate | 0.4687 | 0.1657 to 0.6908 | 0.003 |
|  | ceramide (d18:2/24:1, d18:1/24:2) | 0.505 | 0.2116 to 0.7149 | 0.0012 |
|  | citrate | 0.3833 | 0.06275 to 0.6322 | 0.0175 |
|  | cysteine s-sulfate | 0.3686 | 0.04573 to 0.6218 | 0.0228 |
|  | cytosine | -0.332 | -0.5954 to -0.003824 | 0.0418 |
|  | dopamine 4-sulfate | -0.45 | -0.678 to -0.1422 | 0.0046 |
|  | glutarate (pentanedioate) | 0.3382 | 0.01099 to 0.6 | 0.0378 |
|  | glycodeoxycholate | -0.381 | -0.6309 to -0.06058 | 0.0181 |
|  | hippurate | 0.3247 | -0.004249 to 0.5902 | 0.0467 |
|  | isovalerate | 0.4877 | 0.1896 to 0.7035 | 0.0019 |
|  | malonylcarnitine | -0.336 | -0.5987 to -0.008948 | 0.0389 |
|  | methylphosphate | 0.4232 | 0.11 to 0.6599 | 0.0081 |
|  | N2,N2-dimethylguanosine | 0.3912 | 0.07198 to 0.6377 | 0.0152 |
|  | N-acetylasparagine | 0.352 | 0.02664 to 0.6099 | 0.0302 |
|  | N-acetylcarnosine | 0.4047 | 0.088 to 0.6472 | 0.0117 |
|  | N-acetylisoleucine | 0.5053 | 0.2121 to 0.7151 | 0.0012 |
|  | N-acetylserine | 0.3205 | -0.00889 to 0.5871 | 0.0498 |
|  | N-acetylvaline | 0.3396 | 0.01252 to 0.601 | 0.037 |
|  | N-palmitoyl-sphingosine (d18:1/16:0) | 0.3404 | 0.01347 to 0.6016 | 0.0365 |
|  | o-cresol sulfate | 0.3336 | 0.005797 to 0.5967 | 0.0407 |
|  | oleoyl-linolenoyl-glycerol (18:1/18:3) [2] | 0.4715 | 0.1693 to 0.6927 | 0.0028 |
|  | oleoyl-oleoyl-glycerol (18:1/18:1) [2] | 0.3446 | 0.01818 to 0.6046 | 0.0341 |
|  | oleoyl-oleoyl-glycerol (18:1/18:1)[1] | 0.3413 | 0.01446 to 0.6022 | 0.036 |
|  | O-methylcatechol sulfate | 0.3982 | 0.08023 to 0.6426 | 0.0133 |
|  | pro-hydroxy-pro | 0.5234 | 0.2354 to 0.7269 | 0.0007 |
|  | pyridoxal | 0.4694 | 0.1666 to 0.6913 | 0.0029 |
|  | pyroglutamine | 0.4185 | 0.1044 to 0.6567 | 0.0089 |
|  | sarcosine | -0.355 | -0.6122 to -0.03028 | 0.0286 |
|  | S-methylmethionine | 0.3248 | -0.004063 to 0.5903 | 0.0466 |
|  | sphingomyelin (d18:1/14:0, d16:1/16:0) | -0.339 | -0.6008 to -0.01223 | 0.0372 |
|  | sphingomyelin (d18:1/20:0, d16:1/22:0) | -0.364 | -0.6185 to -0.04043 | 0.0246 |
|  | stearoyl sphingomyelin (d18:1/18:0) | -0.368 | -0.6215 to -0.04522 | 0.0229 |
|  | stearoylcholine | 0.4158 | 0.1012 to 0.6548 | 0.0094 |
|  | succinate | 0.3958 | 0.07739 to 0.6409 | 0.0139 |
|  | tauro-beta-muricholate | -0.322 | -0.5885 to 0.006843 | 0.0484 |
|  | valylglutamine | 0.3458 | 0.01963 to 0.6055 | 0.0334 |
| Vitamin B12 | 2-aminobutyrate | -0.377 | -0.6277 to -0.05536 | 0.0197 |
|  | 4-guanidinobutanoate | -0.47 | -0.6914 to -0.1668 | 0.0029 |
|  | 5-bromotryptophan | 0.3489 | 0.02315 to 0.6077 | 0.0318 |
|  | 5-methylthioadenosine (MTA) | -0.341 | -0.6018 to -0.01371 | 0.0364 |
|  | arabonate/xylonate | 0.3645 | 0.04093 to 0.6188 | 0.0245 |
|  | ascorbate (Vitamin C) | 0.3535 | 0.02839 to 0.611 | 0.0294 |
|  | cysteine-glutathione disulfide | 0.3682 | 0.04522 to 0.6215 | 0.0229 |
|  | cytosine | -0.365 | -0.6189 to -0.041 | 0.0244 |
|  | glycodeoxycholate sulfate | -0.368 | -0.621 to -0.0445 | 0.0232 |
|  | indolepropionylglycine | -0.329 | -0.5933 to -0.0005593 | 0.0437 |
|  | N6,N6,N6-trimethyllysine | -0.357 | -0.6137 to -0.03264 | 0.0277 |
|  | N-formylphenylalanine | 0.3213 | -0.007993 to 0.5877 | 0.0492 |
|  | o-cresol sulfate | 0.3216 | -0.007658 to 0.5879 | 0.049 |
|  | perfluorooctanesulfonic acid (PFOS) | 0.3614 | 0.03741 to 0.6167 | 0.0258 |
|  | pyridoxal | 0.3982 | 0.08025 to 0.6426 | 0.0133 |
|  | succinate | 0.329 | 0.000651 to 0.5934 | 0.0437 |
|  | tauro-beta-muricholate | -0.447 | -0.6762 to -0.1389 | 0.0049 |
|  | threonate | 0.3969 | 0.07868 to 0.6417 | 0.0136 |
| Vitamin B2 | 2-aminobutyrate | -0.336 | -0.5981 to -0.008031 | 0.0394 |
|  | 2-methoxyresorcinol sulfate | 0.3537 | 0.02856 to 0.6111 | 0.0294 |
|  | 3-hydroxyisobutyrate | -0.329 | -0.5932 to -0.0004056 | 0.0438 |
|  | 3-hydroxypyridine sulfate | 0.3986 | 0.08074 to 0.6429 | 0.0132 |
|  | 3-methylcytidine | 0.4465 | 0.1384 to 0.6759 | 0.0049 |
|  | 4-cholesten-3-one | 0.3448 | 0.01842 to 0.6048 | 0.034 |
|  | 5alpha-androstan-3beta,17beta-diol disulfate | 0.3304 | 0.002144 to 0.5943 | 0.0428 |
|  | 5-hydroxydecanoate | 0.3292 | 0.0008965 to 0.5935 | 0.0435 |
|  | arabitol/xylitol | 0.3776 | 0.05612 to 0.6282 | 0.0194 |
|  | arabonate/xylonate | 0.3573 | 0.03264 to 0.6137 | 0.0277 |
|  | catechol sulfate | 0.4735 | 0.1717 to 0.694 | 0.0027 |
|  | ceramide (d18:2/24:1, d18:1/24:2) | 0.5433 | 0.2615 to 0.7397 | 0.0004 |
|  | dopamine 4-sulfate | -0.464 | -0.6876 to -0.1598 | 0.0033 |
|  | hippurate | 0.3446 | 0.01818 to 0.6046 | 0.0341 |
|  | N2,N2-dimethylguanosine | 0.3249 | -0.004005 to 0.5903 | 0.0466 |
|  | N-acetylisoleucine | 0.3273 | -0.001291 to 0.5921 | 0.0449 |
|  | N-behenoyl-sphingadienine (d18:2/22:0) | 0.3888 | 0.06916 to 0.636 | 0.0159 |
|  | N-methylpipecolate | -0.391 | -0.6377 to -0.07198 | 0.0152 |
|  | N-nervonoyl-hexadecasphingosine (d16:1/24:1) | 0.3251 | -0.00376 to 0.5905 | 0.0464 |
|  | o-cresol sulfate | 0.4495 | 0.142 to 0.6778 | 0.0046 |
|  | oleoyl-linolenoyl-glycerol (18:1/18:3) [2] | 0.3633 | 0.03959 to 0.618 | 0.025 |
|  | oleoyl-oleoyl-glycerol (18:1/18:1) [2] | 0.35 | 0.02439 to 0.6085 | 0.0312 |
|  | oleoyl-oleoyl-glycerol (18:1/18:1)[1] | 0.3336 | 0.005813 to 0.5967 | 0.0407 |
|  | O-methylcatechol sulfate | 0.4295 | 0.1176 to 0.6642 | 0.0071 |
|  | pro-hydroxy-pro | 0.4601 | 0.1551 to 0.685 | 0.0037 |
|  | pyridoxal | 0.4771 | 0.1762 to 0.6964 | 0.0025 |
|  | pyroglutamine | 0.3689 | 0.04598 to 0.622 | 0.0227 |
|  | stearoyl sphingomyelin (d18:1/18:0) | -0.378 | -0.6282 to -0.05612 | 0.0194 |
|  | stearoylcholine | 0.405 | 0.08829 to 0.6473 | 0.0117 |
|  | succinate | 0.3203 | -0.009134 to 0.587 | 0.05 |
|  | tauro-beta-muricholate | -0.373 | -0.6247 to -0.05052 | 0.0212 |
|  | tauroursodeoxycholate | -0.367 | -0.6209 to -0.04424 | 0.0233 |
|  | trans-4-hydroxyproline | 0.3352 | 0.007538 to 0.5978 | 0.0397 |
| Vitamin B3 | 2-methoxyresorcinol sulfate | 0.431 | 0.1195 to 0.6653 | 0.0069 |
|  | 3-(3-hydroxyphenyl)propionate | 0.3509 | 0.02533 to 0.6091 | 0.0308 |
|  | 3-(3-hydroxyphenyl)propionate sulfate | 0.4312 | 0.1197 to 0.6654 | 0.0069 |
|  | 3-hydroxyhippurate | 0.442 | 0.1329 to 0.6728 | 0.0055 |
|  | 3-methoxycatechol sulfate (1) | 0.3963 | 0.07803 to 0.6413 | 0.0138 |
|  | 3-methylcytidine | 0.3349 | 0.007292 to 0.5976 | 0.0398 |
|  | 5alpha-androstan-3alpha,17beta-diol disulfate | 0.414 | 0.09899 to 0.6536 | 0.0098 |
|  | adenosine 3',5'-cyclic monophosphate (cAMP) | 0.3301 | 0.001899 to 0.5942 | 0.0429 |
|  | adrenate (22:4n6) | -0.332 | -0.5957 to -0.004337 | 0.0415 |
|  | arabitol/xylitol | 0.3334 | 0.005567 to 0.5965 | 0.0408 |
|  | catechol sulfate | 0.4306 | 0.1189 to 0.665 | 0.007 |
|  | ceramide (d18:2/24:1, d18:1/24:2) | 0.4796 | 0.1794 to 0.6981 | 0.0023 |
|  | cysteine | 0.3275 | -0.001066 to 0.5922 | 0.0447 |
|  | diacylglycerol (12:0/18:1, 14:0/16:1, 16:0/14:1) [1] | 0.3293 | 0.0009436 to 0.5935 | 0.0435 |
|  | glycodeoxycholate | -0.376 | -0.6272 to -0.05463 | 0.0199 |
|  | imidazole propionate | 0.3452 | 0.01885 to 0.605 | 0.0338 |
|  | inosine | -0.352 | -0.6102 to -0.02704 | 0.03 |
|  | malonylcarnitine | -0.331 | -0.5947 to -0.002773 | 0.0424 |
|  | methylphosphate | 0.3304 | 0.002144 to 0.5943 | 0.0428 |
|  | N-acetylasparagine | 0.4608 | 0.1559 to 0.6855 | 0.0036 |
|  | N-acetylisoleucine | 0.3747 | 0.05272 to 0.6261 | 0.0205 |
|  | N-nervonoyl-hexadecasphingosine (d16:1/24:1) | 0.3527 | 0.02739 to 0.6104 | 0.0299 |
|  | o-cresol sulfate | 0.3484 | 0.02256 to 0.6074 | 0.032 |
|  | oleoyl-linolenoyl-glycerol (18:1/18:3) [2] | 0.3632 | 0.03947 to 0.6179 | 0.025 |
|  | O-methylcatechol sulfate | 0.3765 | 0.05485 to 0.6274 | 0.0198 |
|  | pro-hydroxy-pro | 0.3284 | -8.503e-005 to 0.5929 | 0.0441 |
|  | pyridoxal | 0.4188 | 0.1047 to 0.6569 | 0.0089 |
|  | pyroglutamine | 0.3205 | -0.00889 to 0.5871 | 0.0498 |
|  | S-methylmethionine | 0.3745 | 0.0525 to 0.6259 | 0.0205 |
|  | tauro-beta-muricholate | -0.357 | -0.6135 to -0.03228 | 0.0278 |
| Vitamin B6 | 1-(1-enyl-palmitoyl)-2-arachidonoyl-GPE (P-16:0/20:4) | -0.347 | -0.6062 to -0.02068 | 0.0329 |
|  | 1,2,3-benzenetriol sulfate (2) | 0.4065 | 0.09006 to 0.6484 | 0.0113 |
|  | 13-HODE + 9-HODE | -0.339 | -0.6003 to -0.01151 | 0.0375 |
|  | 2-aminophenol sulfate | 0.3849 | 0.06464 to 0.6333 | 0.017 |
|  | 2-methoxyresorcinol sulfate | 0.4614 | 0.1567 to 0.6859 | 0.0036 |
|  | 2'-O-methylcytidine | 0.3943 | 0.07569 to 0.6399 | 0.0143 |
|  | 3-(3-hydroxyphenyl)propionate | 0.3647 | 0.04116 to 0.619 | 0.0244 |
|  | 3-(3-hydroxyphenyl)propionate sulfate | 0.3486 | 0.02274 to 0.6075 | 0.032 |
|  | 3-hydroxyhippurate | 0.4493 | 0.1418 to 0.6777 | 0.0047 |
|  | 3-hydroxylaurate | 0.3895 | 0.06995 to 0.6365 | 0.0157 |
|  | 3-hydroxymyristate | 0.3274 | -0.001168 to 0.5922 | 0.0448 |
|  | 3-methoxycatechol sulfate (1) | 0.3735 | 0.0513 to 0.6252 | 0.0209 |
|  | 3-methylcytidine | 0.4429 | 0.1339 to 0.6734 | 0.0054 |
|  | 3-methylglutarate/2-methylglutarate | 0.3325 | 0.004562 to 0.5959 | 0.0414 |
|  | 5alpha-androstan-3alpha,17beta-diol disulfate | 0.43 | 0.1182 to 0.6646 | 0.0071 |
|  | 5alpha-androstan-3beta,17beta-diol disulfate | 0.3344 | 0.006655 to 0.5972 | 0.0402 |
|  | 5alpha-androstan-3beta,17beta-diol monosulfate (2) | 0.3589 | 0.03453 to 0.6149 | 0.0269 |
|  | arabitol/xylitol | 0.3843 | 0.06393 to 0.6329 | 0.0172 |
|  | behenoyl dihydrosphingomyelin (d18:0/22:0) | 0.3468 | 0.02068 to 0.6062 | 0.0329 |
|  | carboxyibuprofen | 0.3623 | 0.03845 to 0.6173 | 0.0254 |
|  | catechol sulfate | 0.4872 | 0.189 to 0.7031 | 0.0019 |
|  | ceramide (d18:2/24:1, d18:1/24:2) | 0.4772 | 0.1764 to 0.6965 | 0.0024 |
|  | citrate | 0.3928 | 0.07393 to 0.6389 | 0.0147 |
|  | dimethylglycine | 0.3459 | 0.01969 to 0.6056 | 0.0334 |
|  | docosahexaenoate (DHA; 22:6n3) | 0.3266 | -0.002026 to 0.5916 | 0.0453 |
|  | dopamine 3-O-sulfate | 0.3353 | 0.007682 to 0.5979 | 0.0396 |
|  | gamma-glutamyltryptophan | 0.3755 | 0.05363 to 0.6266 | 0.0202 |
|  | guanidinoacetate | 0.3562 | 0.03141 to 0.6129 | 0.0282 |
|  | imidazole propionate | 0.3916 | 0.07251 to 0.638 | 0.015 |
|  | linoleoylcholine | 0.3983 | 0.08039 to 0.6427 | 0.0133 |
|  | N-acetylasparagine | 0.4355 | 0.1249 to 0.6684 | 0.0063 |
|  | N-acetylcarnosine | 0.3671 | 0.04398 to 0.6207 | 0.0234 |
|  | N-acetylisoleucine | 0.3562 | 0.03143 to 0.6129 | 0.0282 |
|  | N-acetylmethionine | 0.3449 | 0.01851 to 0.6048 | 0.034 |
|  | N-acetylvaline | 0.3557 | 0.0308 to 0.6125 | 0.0284 |
|  | N-methylpipecolate | -0.327 | -0.5922 to 0.001168 | 0.0448 |
|  | N-nervonoyl-hexadecasphingosine (d16:1/24:1) | 0.41 | 0.09427 to 0.6508 | 0.0106 |
|  | N-palmitoyl-sphingosine (d18:1/16:0) | 0.3961 | 0.0778 to 0.6412 | 0.0138 |
|  | oleoylcholine | 0.3442 | 0.01773 to 0.6043 | 0.0344 |
|  | oleoyl-linolenoyl-glycerol (18:1/18:3) [2] | 0.4315 | 0.1201 to 0.6656 | 0.0068 |
|  | O-methylcatechol sulfate | 0.5045 | 0.211 to 0.7145 | 0.0012 |
|  | palmitoleoyl-oleoyl-glycerol (16:1/18:1) [2] | 0.3441 | 0.0177 to 0.6043 | 0.0344 |
|  | palmitoylcholine | 0.3377 | 0.01039 to 0.5996 | 0.0381 |
|  | picolinate | 0.3828 | 0.06214 to 0.6318 | 0.0177 |
|  | pyridoxal | 0.4069 | 0.09056 to 0.6487 | 0.0112 |
|  | pyroglutamine | 0.4769 | 0.176 to 0.6963 | 0.0025 |
|  | sedoheptulose | -0.389 | -0.6361 to -0.06926 | 0.0158 |
|  | S-methylmethionine | 0.3348 | 0.007134 to 0.5975 | 0.0399 |
|  | spermidine | -0.321 | -0.5878 to 0.00794 | 0.0492 |
|  | stearoylcholine | 0.4348 | 0.124 to 0.6679 | 0.0064 |
|  | taurine | -0.401 | -0.6443 to -0.0831 | 0.0127 |
|  | tauro-beta-muricholate | -0.365 | -0.6193 to -0.04174 | 0.0242 |
|  | thioproline | 0.3343 | 0.006573 to 0.5972 | 0.0402 |
| Vitamin C | 1-linoleoyl-GPG (18:2) | 0.324 | -0.004983 to 0.5897 | 0.0472 |
|  | 2-aminobutyrate | -0.33 | -0.5938 to -0.001387 | 0.0432 |
|  | 3-indoxyl sulfate | -0.356 | -0.6129 to -0.03139 | 0.0282 |
|  | 4-acetylphenol sulfate | 0.3579 | 0.03334 to 0.6141 | 0.0274 |
|  | allantoin | -0.436 | -0.6687 to -0.1256 | 0.0062 |
|  | argininate | -0.341 | -0.6022 to -0.01446 | 0.036 |
|  | C-glycosyltryptophan | -0.321 | -0.5873 to 0.008646 | 0.0496 |
|  | creatine | -0.34 | -0.6013 to -0.01297 | 0.0368 |
|  | cysteine-glutathione disulfide | -0.405 | -0.647 to -0.08774 | 0.0118 |
|  | erythronate | -0.34 | -0.601 to -0.01248 | 0.037 |
|  | linolenate [alpha or gamma; (18:3n3 or 6)] | 0.3317 | 0.003599 to 0.5953 | 0.0419 |
|  | N2-acetyllysine | -0.343 | -0.6036 to -0.01662 | 0.0349 |
|  | N-acetylneuraminate | -0.343 | -0.6033 to -0.01619 | 0.0351 |
|  | N-formylphenylalanine | -0.405 | -0.6472 to -0.08804 | 0.0117 |
|  | ophthalmate | -0.354 | -0.6115 to -0.02911 | 0.0291 |
|  | phenol sulfate | -0.567 | -0.7545 to -0.2924 | 0.0002 |
|  | propionylcarnitine (C3) | -0.353 | -0.6106 to -0.02764 | 0.0298 |
|  | propionylglycine | -0.472 | -0.693 to -0.1699 | 0.0028 |
|  | uracil | -0.342 | -0.6029 to -0.01554 | 0.0354 |
|  | urea | -0.363 | -0.6178 to -0.03917 | 0.0251 |
| Vitamin D | 1-(1-enyl-palmitoyl)-2-oleoyl-GPE (P-16:0/18:1) | 0.3387 | 0.01149 to 0.6003 | 0.0375 |
|  | 1-(1-enyl-stearoyl)-2-oleoyl-GPE (P-18:0/18:1) | 0.3225 | -0.006694 to 0.5886 | 0.0483 |
|  | 1-arachidonoyl-GPC (20:4n6) | 0.3636 | 0.03993 to 0.6182 | 0.0248 |
|  | 1-oleoyl-GPC (18:1) | 0.3774 | 0.05587 to 0.628 | 0.0195 |
|  | 1-oleoyl-GPE (18:1) | 0.3299 | 0.001633 to 0.594 | 0.0431 |
|  | 1-palmitoleoyl-GPC (16:1) | 0.3645 | 0.04093 to 0.6188 | 0.0245 |
|  | 3-hydroxypyridine sulfate | 0.3454 | 0.01917 to 0.6052 | 0.0337 |
|  | 4-guanidinobutanoate | -0.324 | -0.5897 to 0.004983 | 0.0472 |
|  | 5-bromotryptophan | 0.4111 | 0.09555 to 0.6516 | 0.0103 |
|  | arabonate/xylonate | 0.4047 | 0.088 to 0.6472 | 0.0117 |
|  | campesterol | 0.3473 | 0.02124 to 0.6065 | 0.0327 |
|  | ceramide (d18:2/24:1, d18:1/24:2) | 0.4476 | 0.1397 to 0.6766 | 0.0048 |
|  | cholesterol | 0.3428 | 0.01619 to 0.6033 | 0.0351 |
|  | cytosine | -0.343 | -0.6033 to -0.01612 | 0.0352 |
|  | eicosanodioate | 0.3402 | 0.01322 to 0.6014 | 0.0366 |
|  | glycosyl ceramide (d18:2/24:1, d18:1/24:2) | 0.5111 | 0.2195 to 0.7189 | 0.001 |
|  | glycosyl-N-nervonoyl-sphingosine (d18:1/24:1) | 0.4336 | 0.1227 to 0.6671 | 0.0065 |
|  | glycosyl-N-palmitoyl-sphingosine (d18:1/16:0) | 0.3395 | 0.01248 to 0.601 | 0.037 |
|  | lactosyl-N-nervonoyl-sphingosine (d18:1/24:1) | 0.4183 | 0.1042 to 0.6566 | 0.009 |
|  | lactosyl-N-palmitoyl-sphingosine (d18:1/16:0) | 0.4317 | 0.1203 to 0.6657 | 0.0068 |
|  | N6-carbamoylthreonyladenosine | 0.3309 | 0.002697 to 0.5947 | 0.0425 |
|  | nervonoylcarnitine (C24:1) | 0.3443 | 0.01793 to 0.6044 | 0.0343 |
|  | N-methylpipecolate | -0.378 | -0.6285 to -0.05663 | 0.0193 |
|  | o-cresol sulfate | 0.3984 | 0.08046 to 0.6427 | 0.0132 |
|  | oleoylcholine | 0.3213 | -0.007993 to 0.5877 | 0.0492 |
|  | sphingomyelin (d18:1/24:1, d18:2/24:0) | 0.3378 | 0.0105 to 0.5997 | 0.0381 |
|  | sphingomyelin (d18:2/24:1, d18:1/24:2) | 0.3667 | 0.04345 to 0.6204 | 0.0236 |
|  | stearoylcholine | 0.4232 | 0.1101 to 0.6599 | 0.0081 |
| Vitamin E | 10-heptadecenoate (17:1n7) | -0.459 | -0.6841 to -0.1534 | 0.0038 |
|  | 1-arachidonoyl-GPI (20:4) | -0.332 | -0.5957 to -0.004234 | 0.0416 |
|  | 1-methylhistidine | -0.357 | -0.6137 to -0.03266 | 0.0277 |
|  | 2-aminobutyrate | -0.389 | -0.6361 to -0.06931 | 0.0158 |
|  | 2-aminoheptanoate | 0.455 | 0.1488 to 0.6816 | 0.0041 |
|  | 2-aminooctanoate | 0.4146 | 0.09976 to 0.654 | 0.0096 |
|  | 2-hydroxybutyrate/2-hydroxyisobutyrate | -0.397 | -0.6415 to -0.07832 | 0.0137 |
|  | 2-hydroxystearate | -0.363 | -0.6179 to -0.03945 | 0.025 |
|  | 3-aminoisobutyrate | -0.392 | -0.6382 to -0.07278 | 0.015 |
|  | 3-hydroxy-2-ethylpropionate | -0.372 | -0.6242 to -0.04967 | 0.0214 |
|  | 3-hydroxybutyrate (BHBA) | -0.344 | -0.6041 to -0.01745 | 0.0345 |
|  | 3-hydroxybutyrylcarnitine (1) | -0.407 | -0.6489 to -0.09099 | 0.0112 |
|  | 3-hydroxybutyrylcarnitine (2) | -0.494 | -0.7079 to -0.1981 | 0.0016 |
|  | 3-hydroxydecanoate | -0.435 | -0.6678 to -0.124 | 0.0064 |
|  | 3-hydroxylaurate | -0.48 | -0.6981 to -0.1794 | 0.0023 |
|  | 3-hydroxymyristate | -0.4 | -0.644 to -0.08258 | 0.0128 |
|  | 3-hydroxyoctanoate | -0.327 | -0.5916 to 0.002026 | 0.0453 |
|  | 5-methyluridine (ribothymidine) | -0.328 | -0.5923 to 0.001026 | 0.0447 |
|  | acetoacetate | -0.367 | -0.6204 to -0.04339 | 0.0236 |
|  | acetylcarnitine (C2) | -0.438 | -0.6699 to -0.1277 | 0.006 |
|  | arabonate/xylonate | 0.3833 | 0.06278 to 0.6322 | 0.0175 |
|  | arachidonoyl ethanolamide | -0.398 | -0.6424 to -0.07997 | 0.0133 |
|  | ceramide (d18:1/14:0, d16:1/16:0) | -0.385 | -0.6332 to -0.06444 | 0.0171 |
|  | decanoylcarnitine (C10) | -0.408 | -0.6497 to -0.09232 | 0.0109 |
|  | deoxycarnitine | -0.462 | -0.6862 to -0.1572 | 0.0035 |
|  | docosadienoate (22:2n6) | -0.34 | -0.6011 to -0.01262 | 0.0369 |
|  | docosapentaenoate (n3 DPA; 22:5n3) | -0.35 | -0.6088 to -0.02479 | 0.031 |
|  | gamma-glutamylleucine | -0.409 | -0.6503 to -0.09336 | 0.0107 |
|  | gamma-glutamylvaline | -0.444 | -0.6744 to -0.1357 | 0.0052 |
|  | hexanoylcarnitine (C6) | -0.46 | -0.6846 to -0.1543 | 0.0037 |
|  | hexanoylglycine | -0.36 | -0.6153 to -0.03525 | 0.0266 |
|  | hydantoin-5-propionic acid | -0.34 | -0.601 to -0.01258 | 0.037 |
|  | ibuprofen | -0.371 | -0.6231 to -0.04788 | 0.022 |
|  | indole-3-carboxylic acid | -0.431 | -0.6651 to -0.1192 | 0.0069 |
|  | indolepropionate | 0.357 | 0.03229 to 0.6135 | 0.0278 |
|  | kynurenate | -0.486 | -0.7024 to -0.1876 | 0.002 |
|  | laurylcarnitine (C12) | -0.602 | -0.7767 to -0.3406 | <0.0001 |
|  | leucine | -0.352 | -0.6101 to -0.02691 | 0.0301 |
|  | linoleoyl ethanolamide | -0.341 | -0.6019 to -0.01388 | 0.0363 |
|  | linoleoyl-linolenoyl-glycerol (18:2/18:3) [2] | 0.4776 | 0.177 to 0.6968 | 0.0024 |
|  | margarate (17:0) | -0.346 | -0.6053 to -0.01931 | 0.0336 |
|  | methyl-4-hydroxybenzoate sulfate | -0.422 | -0.6593 to -0.109 | 0.0083 |
|  | myristate (14:0) | -0.359 | -0.6147 to -0.03429 | 0.027 |
|  | myristoleate (14:1n5) | -0.44 | -0.6711 to -0.1299 | 0.0058 |
|  | myristoleoylcarnitine (C14:1) | -0.536 | -0.7352 to -0.2523 | 0.0005 |
|  | myristoylcarnitine (C14) | -0.541 | -0.7384 to -0.2588 | 0.0005 |
|  | N6,N6,N6-trimethyllysine | -0.343 | -0.6035 to -0.01646 | 0.035 |
|  | N-acetyl-1-methylhistidine | -0.455 | -0.6816 to -0.1488 | 0.0041 |
|  | N-acetylarginine | -0.394 | -0.6399 to -0.07574 | 0.0143 |
|  | N-acetylcitrulline | -0.337 | -0.5993 to -0.009817 | 0.0384 |
|  | N-acetylleucine | -0.383 | -0.6319 to -0.0624 | 0.0176 |
|  | N-acetylmethionine | -0.326 | -0.5911 to 0.002785 | 0.0458 |
|  | N-acetylneuraminate | -0.363 | -0.6178 to -0.03932 | 0.0251 |
|  | N-delta-acetylornithine | 0.3361 | 0.008545 to 0.5984 | 0.0391 |
|  | N-methylpipecolate | -0.351 | -0.609 to -0.02516 | 0.0309 |
|  | octadecanedioate | -0.323 | -0.5891 to 0.005819 | 0.0478 |
|  | octanoylcarnitine (C8) | -0.38 | -0.6295 to -0.05831 | 0.0188 |
|  | oleate/vaccenate (18:1) | -0.415 | -0.6539 to -0.09962 | 0.0097 |
|  | oleoyl ethanolamide | -0.373 | -0.6249 to -0.05081 | 0.0211 |
|  | oleoyl-arachidonoyl-glycerol (18:1/20:4) [1] | 0.3649 | 0.04146 to 0.6192 | 0.0243 |
|  | oleoylcarnitine (C18:1) | -0.354 | -0.6117 to -0.02941 | 0.029 |
|  | palmitate (16:0) | -0.362 | -0.617 to -0.03794 | 0.0256 |
|  | palmitoleate (16:1n7) | -0.422 | -0.6588 to -0.1082 | 0.0084 |
|  | palmitoleoylcarnitine (C16:1) | -0.496 | -0.7087 to -0.1996 | 0.0016 |
|  | propyl 4-hydroxybenzoate sulfate | -0.338 | -0.5998 to -0.01061 | 0.038 |
|  | S-1-pyrroline-5-carboxylate | 0.3254 | -0.003419 to 0.5907 | 0.0462 |
|  | sphingomyelin (d18:1/14:0, d16:1/16:0) | -0.363 | -0.6178 to -0.03932 | 0.0251 |
|  | sphingomyelin (d18:1/15:0, d16:1/17:0) | -0.358 | -0.6143 to -0.03367 | 0.0273 |
|  | sphingomyelin (d18:1/18:1, d18:2/18:0) | -0.345 | -0.6048 to -0.01857 | 0.0339 |
|  | stearoyl ethanolamide | -0.432 | -0.666 to -0.1207 | 0.0068 |
|  | taurocholenate sulfate | -0.421 | -0.6585 to -0.1075 | 0.0085 |
|  | uridine | -0.468 | -0.6901 to -0.1645 | 0.0031 |
