# Supplementary material for: Plasma metabolomics of children with aberrant serum lipids and inadequate micronutrient intake
Source: PLoS One. 2018 Oct 31;13(10):e0205899. doi: 10.1371/journal.pone.0205899 (PMC6209210; doi:10.1371/journal.pone.0205899)
Supplement: S5 Table — (DOCX) [file pone.0205899.s010.docx]

| **S5 Table Plasma Metabolites Significantly Correlated with Mineral Status in Children** | | | | |
| --- | --- | --- | --- | --- |
| **Micronutrient** | **Biochemical Name** | **Spearman’s Correlation Coefficient** | | **P-value** |
|  |  | **r_s_** | **95% CI** |  |
| Calcium | 2-aminobutyrate | 0.3345 | 0.006799 to 0.5973 | 0.0401 |
|  | 2-aminooctanoate | -0.3378 | -0.5997 to -0.0105 | 0.0381 |
|  | 3-phenylpropionate (hydrocinnamate) | -0.3376 | -0.5995 to -0.01025 | 0.0382 |
|  | arabonate/xylonate | -0.3732 | -0.6251 to -0.05104 | 0.021 |
|  | bilirubin (E,Z or Z,E) | 0.3616 | 0.03766 to 0.6168 | 0.0257 |
|  | biliverdin | 0.352 | 0.02664 to 0.6099 | 0.0302 |
|  | campesterol | -0.3394 | -0.6009 to -0.01231 | 0.0371 |
|  | chenodeoxycholate | -0.3472 | -0.6065 to -0.02118 | 0.0327 |
|  | creatine | 0.3619 | 0.03791 to 0.617 | 0.0256 |
|  | dehydroisoandrosterone sulfate (DHEA-S) | 0.3503 | 0.02464 to 0.6087 | 0.0311 |
|  | docosadioate | -0.3544 | -0.6117 to -0.02939 | 0.029 |
|  | eicosanodioate | -0.4078 | -0.6493 to -0.09164 | 0.011 |
|  | galactonate | -0.3276 | -0.5923 to 0.0009705 | 0.0447 |
|  | gamma-glutamylvaline | 0.3428 | 0.01619 to 0.6033 | 0.0351 |
|  | glycodeoxycholate sulfate | 0.4668 | 0.1634 to 0.6895 | 0.0031 |
|  | glycolithocholate sulfate | 0.3851 | 0.0648 to 0.6334 | 0.017 |
|  | hippurate | -0.4015 | -0.6449 to -0.08411 | 0.0125 |
|  | hyocholate | -0.3327 | -0.5961 to -0.004829 | 0.0412 |
|  | L-urobilin | -0.4858 | -0.7022 to -0.1873 | 0.002 |
|  | malonylcarnitine | 0.3289 | 0.0005559 to 0.5933 | 0.0437 |
|  | N-acetylarginine | 0.3579 | 0.03339 to 0.6142 | 0.0274 |
|  | N-acetylaspartate (NAA) | -0.3406 | -0.6018 to -0.01371 | 0.0364 |
|  | N-methylpipecolate | 0.4614 | 0.1567 to 0.6859 | 0.0035 |
|  | o-cresol sulfate | -0.3564 | -0.6131 to -0.03165 | 0.0281 |
|  | orotidine | -0.3332 | -0.5964 to -0.005321 | 0.0409 |
|  | ribonate | -0.3678 | -0.6212 to -0.04472 | 0.0231 |
| Iron | 16-hydroxypalmitate | -0.3279 | -0.5926 to 0.0005755 | 0.0444 |
|  | 2-acetamidophenol sulfate | 0.344 | 0.01752 to 0.6042 | 0.0345 |
|  | 3-(3-hydroxyphenyl)propionate | 0.4192 | 0.1053 to 0.6572 | 0.0088 |
|  | 3-hydroxyhippurate | 0.3391 | 0.01203 to 0.6007 | 0.0373 |
|  | 3-methoxycatechol sulfate (1) | 0.3672 | 0.04407 to 0.6208 | 0.0233 |
|  | 4-vinylphenol sulfate | 0.4409 | 0.1314 to 0.672 | 0.0056 |
|  | 6-oxopiperidine-2-carboxylate | 0.3213 | -0.008008 to 0.5877 | 0.0492 |
|  | adenosine 5'-diphosphoribose (ADP-ribose) | -0.3704 | -0.623 to -0.04772 | 0.0221 |
|  | allantoin | -0.3717 | -0.624 to -0.04927 | 0.0216 |
|  | ceramide (d18:1/17:0, d17:1/18:0) | -0.4689 | -0.6909 to -0.166 | 0.003 |
|  | Cetirizine | 0.3209 | -0.008459 to 0.5874 | 0.0495 |
|  | decanoylcarnitine (C10) | 0.3257 | -0.003026 to 0.591 | 0.046 |
|  | erucate (22:1n9) | -0.4352 | -0.6681 to -0.1245 | 0.0063 |
|  | erythronate | -0.3724 | -0.6244 to -0.05003 | 0.0213 |
|  | flavin adenine dinucleotide (FAD) | -0.3313 | -0.595 to -0.003204 | 0.0422 |
|  | glutamate | -0.3573 | -0.6137 to -0.03264 | 0.0277 |
|  | hexanoylglycine | -0.37 | -0.6227 to -0.04724 | 0.0222 |
|  | inosine 5'-monophosphate (IMP) | -0.3704 | -0.623 to -0.04772 | 0.0221 |
|  | isoursodeoxycholate | 0.3853 | 0.06508 to 0.6336 | 0.0169 |
|  | L-urobilin | -0.39 | -0.6369 to -0.07064 | 0.0155 |
|  | malate | -0.4067 | -0.6485 to -0.09034 | 0.0113 |
|  | margaroylcarnitine | -0.3879 | -0.6354 to -0.06813 | 0.0161 |
|  | N6-succinyladenosine | -0.3282 | -0.5927 to 0.000306 | 0.0443 |
|  | N-acetylaspartate (NAA) | -0.3855 | -0.6337 to -0.06531 | 0.0168 |
|  | N-acetylneuraminate | -0.3347 | -0.5975 to -0.007045 | 0.04 |
|  | N-acetyltaurine | -0.4518 | -0.6794 to -0.1448 | 0.0044 |
|  | N-palmitoyl-sphinganine (d18:0/16:0) | -0.4249 | -0.6611 to -0.1121 | 0.0078 |
|  | orotidine | -0.4693 | -0.6912 to -0.1665 | 0.003 |
|  | p-cresol-glucuronide | -0.325 | -0.5905 to 0.003806 | 0.0465 |
|  | picolinate | 0.3236 | -0.005472 to 0.5894 | 0.0475 |
|  | S-methylcysteine | 0.3343 | 0.006552 to 0.5972 | 0.0402 |
|  | S-methylcysteine sulfoxide | 0.4056 | 0.08904 to 0.6478 | 0.0115 |
|  | stearoylcarnitine (C18) | -0.359 | -0.6149 to -0.03465 | 0.0269 |
|  | taurochenodeoxycholate | -0.3687 | -0.6219 to -0.04582 | 0.0227 |
|  | taurocholate | -0.3259 | -0.591 to 0.002903 | 0.0459 |
|  | taurocholenate sulfate | -0.3851 | -0.6334 to -0.0648 | 0.017 |
|  | taurolithocholate 3-sulfate | -0.3601 | -0.6157 to -0.03593 | 0.0264 |
|  | tyramine O-sulfate | -0.3679 | -0.6213 to -0.0449 | 0.023 |
|  | vanillylmandelate (VMA) | -0.3378 | -0.5997 to -0.0105 | 0.0381 |
| Magnesium | 1-(1-enyl-palmitoyl)-2-arachidonoyl-GPC (P-16:0/20:4) | 0.338 | 0.01075 to 0.5999 | 0.0379 |
|  | 1-(1-enyl-palmitoyl)-2-arachidonoyl-GPE (P-16:0/20:4) | 0.3936 | 0.07481 to 0.6394 | 0.0145 |
|  | 1-linoleoyl-GPI (18:2) | -0.3728 | -0.6247 to -0.05054 | 0.0212 |
|  | 1-oleoyl-GPG (18:1) | -0.3314 | -0.5951 to -0.003311 | 0.0421 |
|  | 2,3-dihydroxy-2-methylbutyrate | 0.3485 | 0.02265 to 0.6074 | 0.032 |
|  | 2-hydroxybutyrate/2-hydroxyisobutyrate | 0.3562 | 0.03139 to 0.6129 | 0.0282 |
|  | 3-methoxytyrosine | -0.4649 | -0.6883 to -0.1611 | 0.0033 |
|  | 4-hydroxychlorothalonil | -0.3233 | -0.5892 to 0.005716 | 0.0477 |
|  | 6-oxopiperidine-2-carboxylate | 0.3896 | 0.07011 to 0.6366 | 0.0156 |
|  | arabonate/xylonate | -0.3763 | -0.6272 to -0.0546 | 0.0199 |
|  | ascorbate (Vitamin C) | -0.3669 | -0.6206 to -0.04371 | 0.0235 |
|  | cysteine-glutathione disulfide | -0.3616 | -0.6168 to -0.03766 | 0.0257 |
|  | eicosanodioate | -0.4271 | -0.6626 to -0.1147 | 0.0075 |
|  | eicosenoylcarnitine (C20:1) | -0.3833 | -0.6322 to -0.06275 | 0.0175 |
|  | erythronate | -0.3925 | -0.6386 to -0.07352 | 0.0148 |
|  | glutarylcarnitine (C5-DC) | 0.4015 | 0.08411 to 0.6449 | 0.0125 |
|  | glycerol | 0.3207 | -0.008646 to 0.5873 | 0.0496 |
|  | glycodeoxycholate sulfate | 0.3595 | 0.03516 to 0.6153 | 0.0267 |
|  | hypotaurine | -0.4098 | -0.6507 to -0.09398 | 0.0106 |
|  | kynurenate | 0.3371 | 0.009758 to 0.5992 | 0.0385 |
|  | malonylcarnitine | 0.4211 | 0.1076 to 0.6585 | 0.0085 |
|  | N6-carbamoylthreonyladenosine | -0.3276 | -0.5923 to 0.0009856 | 0.0447 |
|  | N-oleoyltaurine | -0.3626 | -0.6175 to -0.03876 | 0.0253 |
|  | N-stearoyltaurine | -0.342 | -0.6028 to -0.0153 | 0.0356 |
|  | orotidine | -0.3354 | -0.598 to -0.007785 | 0.0396 |
|  | sarcosine | 0.4101 | 0.0944 to 0.6509 | 0.0105 |
|  | sphingomyelin (d18:1/20:1, d18:2/20:0) | -0.3896 | -0.6366 to -0.07018 | 0.0156 |
| Potassium | 1-(1-enyl-palmitoyl)-2-arachidonoyl-GPE (P-16:0/20:4) | 0.4214 | 0.1079 to 0.6587 | 0.0084 |
|  | 1-methylnicotinamide | 0.3634 | 0.03967 to 0.6181 | 0.0249 |
|  | 1-oleoyl-GPC (18:1) | -0.3327 | -0.5961 to -0.004829 | 0.0412 |
|  | 2-aminoadipate | 0.3486 | 0.02274 to 0.6075 | 0.032 |
|  | 2-hydroxybutyrate/2-hydroxyisobutyrate | 0.4363 | 0.1258 to 0.6689 | 0.0062 |
|  | 2-oxoarginine | 0.328 | -0.0005535 to 0.5926 | 0.0444 |
|  | 3-hydroxydecanoate | 0.3389 | 0.01173 to 0.6005 | 0.0374 |
|  | 3-hydroxyisobutyrate | 0.3498 | 0.02414 to 0.6084 | 0.0313 |
|  | 3-hydroxylaurate | 0.3299 | 0.001633 to 0.594 | 0.0431 |
|  | 3-methoxytyrosine | -0.4284 | -0.6635 to -0.1163 | 0.0073 |
|  | 3-phenylpropionate (hydrocinnamate) | -0.3691 | -0.6221 to -0.04623 | 0.0226 |
|  | acisoga | 0.3277 | -0.0008006 to 0.5924 | 0.0446 |
|  | arabonate/xylonate | -0.38 | -0.6299 to -0.05893 | 0.0186 |
|  | arachidonoyl ethanolamide | 0.3252 | -0.00368 to 0.5905 | 0.0464 |
|  | arachidoylcarnitine (C20) | -0.3949 | -0.6403 to -0.07636 | 0.0141 |
|  | ascorbate (Vitamin C) | -0.347 | -0.6063 to -0.02091 | 0.0328 |
|  | campesterol | -0.3256 | -0.5909 to 0.003191 | 0.0461 |
|  | cysteine-glutathione disulfide | -0.3715 | -0.6238 to -0.04902 | 0.0217 |
|  | eicosanodioate | -0.4757 | -0.6955 to -0.1745 | 0.0025 |
|  | eicosenoylcarnitine (C20:1) | -0.331 | -0.5948 to -0.002861 | 0.0424 |
|  | glutarylcarnitine (C5-DC) | 0.3702 | 0.0475 to 0.6229 | 0.0222 |
|  | glycerol | 0.4404 | 0.1309 to 0.6717 | 0.0057 |
|  | glycine | -0.3253 | -0.5906 to 0.003515 | 0.0463 |
|  | glycodeoxycholate sulfate | 0.3329 | 0.004993 to 0.5962 | 0.0411 |
|  | hypotaurine | -0.4951 | -0.7084 to -0.1991 | 0.0016 |
|  | kynurenate | 0.4124 | 0.09711 to 0.6525 | 0.0101 |
|  | L-urobilin | -0.3532 | -0.6108 to -0.028 | 0.0296 |
|  | malonylcarnitine | 0.3915 | 0.07236 to 0.6379 | 0.0151 |
|  | N6-carbamoylthreonyladenosine | -0.349 | -0.6078 to -0.02324 | 0.0317 |
|  | palmitoyl-arachidonoyl-glycerol (16:0/20:4) [1] | 0.5007 | 0.2062 to 0.7121 | 0.0014 |
|  | ribonate | -0.3549 | -0.612 to -0.02989 | 0.0288 |
|  | sarcosine | 0.4263 | 0.1138 to 0.6621 | 0.0076 |
|  | sphingomyelin (d18:0/18:0, d19:0/17:0) | 0.391 | 0.07172 to 0.6375 | 0.0152 |
|  | sphingomyelin (d18:1/20:1, d18:2/20:0) | -0.3651 | -0.6193 to -0.04169 | 0.0242 |
|  | uridine | 0.3389 | 0.01173 to 0.6005 | 0.0374 |
|  | xanthurenate | 0.3976 | 0.07959 to 0.6422 | 0.0134 |
| Selenium | 1-oleoyl-GPG (18:1) | -0.3984 | -0.6427 to -0.08048 | 0.0132 |
|  | 2,3-dihydroxy-2-methylbutyrate | 0.3686 | 0.04573 to 0.6218 | 0.0228 |
|  | 3-methoxytyrosine | -0.3857 | -0.6339 to -0.06557 | 0.0168 |
|  | 3-ureidopropionate | 0.3732 | 0.05104 to 0.6251 | 0.021 |
|  | 6-oxopiperidine-2-carboxylate | 0.536 | 0.2519 to 0.7351 | 0.0005 |
|  | adenosine 5'-diphosphoribose (ADP-ribose) | -0.3371 | -0.5992 to -0.009772 | 0.0385 |
|  | allantoin | -0.4047 | -0.6472 to -0.088 | 0.0117 |
|  | arabonate/xylonate | -0.3901 | -0.6369 to -0.0707 | 0.0155 |
|  | arachidoylcarnitine (C20) | -0.4019 | -0.6452 to -0.08463 | 0.0124 |
|  | behenoylcarnitine (C22) | -0.329 | -0.5934 to -0.000651 | 0.0437 |
|  | chiro-inositol | 0.3586 | 0.03419 to 0.6147 | 0.027 |
|  | docosadioate | -0.389 | -0.6362 to -0.06941 | 0.0158 |
|  | eicosanodioate | -0.4275 | -0.6629 to -0.1152 | 0.0074 |
|  | eicosenoylcarnitine (C20:1) | -0.3398 | -0.6011 to -0.01272 | 0.0369 |
|  | erythronate | -0.4643 | -0.6878 to -0.1603 | 0.0033 |
|  | inosine 5'-monophosphate (IMP) | -0.3371 | -0.5992 to -0.009772 | 0.0385 |
|  | isovalerylglycine | 0.3436 | 0.01712 to 0.6039 | 0.0347 |
|  | L-urobilin | -0.4286 | -0.6636 to -0.1165 | 0.0073 |
|  | margaroylcarnitine | -0.3349 | -0.5976 to -0.007292 | 0.0398 |
|  | N4-acetylcytidine | -0.3235 | -0.5893 to 0.005574 | 0.0476 |
|  | N6-succinyladenosine | -0.3623 | -0.6173 to -0.03842 | 0.0254 |
|  | N-stearoyltaurine | -0.3447 | -0.6047 to -0.01835 | 0.034 |
|  | orotidine | -0.5065 | -0.7159 to -0.2136 | 0.0012 |
|  | palmitoylcarnitine (C16) | -0.3621 | -0.6171 to -0.03816 | 0.0255 |
|  | prolylglycine | -0.4282 | -0.6633 to -0.116 | 0.0073 |
|  | sarcosine | 0.4015 | 0.08413 to 0.6449 | 0.0125 |
|  | taurine | -0.3498 | -0.6084 to -0.02414 | 0.0313 |
|  | undecanedioate | 0.3255 | -0.00327 to 0.5908 | 0.0461 |
| Sodium | 1-methylhistidine | 0.326 | -0.002781 to 0.5911 | 0.0458 |
|  | 4-guanidinobutanoate | 0.4494 | 0.1419 to 0.6778 | 0.0046 |
|  | 5alpha-androstan-3alpha,17beta-diol monosulfate (1) | 0.3317 | 0.00368 to 0.5953 | 0.0419 |
|  | 5alpha-androstan-3beta,17beta-diol disulfate | 0.3518 | 0.02641 to 0.6098 | 0.0303 |
|  | adrenoylcarnitine (C22:4) | 0.4496 | 0.1422 to 0.6779 | 0.0046 |
|  | androstenediol (3beta,17beta) monosulfate (2) | 0.3404 | 0.01349 to 0.6016 | 0.0365 |
|  | arachidonoylcarnitine (C20:4) | 0.4603 | 0.1554 to 0.6852 | 0.0036 |
|  | arginine | -0.3325 | -0.5959 to -0.004583 | 0.0414 |
|  | betaine | -0.415 | -0.6543 to -0.1003 | 0.0096 |
|  | campesterol | -0.3236 | -0.5894 to 0.005393 | 0.0475 |
|  | Cetirizine | 0.3644 | 0.04086 to 0.6188 | 0.0245 |
|  | corticosterone | -0.3606 | -0.6161 to -0.03644 | 0.0262 |
|  | deoxycholate | 0.3661 | 0.04285 to 0.62 | 0.0238 |
|  | dihomo-linolenoylcarnitine (20:3n3 or 6) | 0.4789 | 0.1786 to 0.6976 | 0.0024 |
|  | dihomo-linoleoylcarnitine (C20:2) | 0.3426 | 0.01594 to 0.6032 | 0.0352 |
|  | docosapentaenoylcarnitine (C22:5n3) | 0.4347 | 0.124 to 0.6678 | 0.0064 |
|  | gamma-glutamyl-2-aminobutyrate | -0.3551 | -0.6121 to -0.03016 | 0.0287 |
|  | gamma-glutamylleucine | 0.3695 | 0.04674 to 0.6224 | 0.0224 |
|  | gamma-glutamylvaline | 0.4511 | 0.144 to 0.679 | 0.0045 |
|  | glycerol | 0.3588 | 0.0344 to 0.6148 | 0.027 |
|  | glycochenodeoxycholate glucuronide (1) | 0.3729 | 0.05061 to 0.6248 | 0.0211 |
|  | glycodeoxycholate sulfate | 0.4593 | 0.1541 to 0.6845 | 0.0037 |
|  | glycolithocholate sulfate | 0.3717 | 0.04927 to 0.624 | 0.0216 |
|  | indoleacetylglutamine | 0.4189 | 0.1049 to 0.657 | 0.0088 |
|  | indolelactate | 0.38 | 0.05893 to 0.6299 | 0.0186 |
|  | indolepropionylglycine | 0.4439 | 0.1352 to 0.6741 | 0.0052 |
|  | kynurenate | 0.4522 | 0.1454 to 0.6797 | 0.0044 |
|  | L-urobilin | -0.3392 | -0.6007 to -0.01214 | 0.0372 |
|  | malonylcarnitine | 0.3441 | 0.01764 to 0.6042 | 0.0344 |
|  | methyl-4-hydroxybenzoate sulfate | 0.4534 | 0.1468 to 0.6805 | 0.0043 |
|  | N6,N6,N6-trimethyllysine | 0.5647 | 0.2901 to 0.7534 | 0.0002 |
|  | N-acetyl-1-methylhistidine | 0.3273 | -0.001284 to 0.5921 | 0.0449 |
|  | ornithine | 0.4183 | 0.1042 to 0.6566 | 0.009 |
|  | palmitoyl-myristoyl-glycerol (16:0/14:0) [2] | 0.3389 | 0.01171 to 0.6005 | 0.0374 |
|  | propyl 4-hydroxybenzoate sulfate | 0.396 | 0.0776 to 0.641 | 0.0139 |
|  | pyruvate | 0.3489 | 0.02315 to 0.6077 | 0.0318 |
|  | retinol (Vitamin A) | 0.4019 | 0.08463 to 0.6452 | 0.0124 |
|  | tryptophan | 0.3476 | 0.02165 to 0.6068 | 0.0325 |
|  | tyramine O-sulfate | -0.3451 | -0.605 to -0.0188 | 0.0338 |
|  | vanillylmandelate (VMA) | -0.324 | -0.5897 to 0.004983 | 0.0472 |
|  | xanthurenate | 0.4464 | 0.1383 to 0.6758 | 0.005 |
| Zinc | 1-methylimidazoleacetate | -0.3868 | -0.6346 to -0.06685 | 0.0164 |
|  | 2-hydroxybutyrate/2-hydroxyisobutyrate | 0.3789 | 0.05765 to 0.6291 | 0.019 |
|  | 3-methoxytyrosine | -0.4409 | -0.672 to -0.1314 | 0.0056 |
|  | 6-oxopiperidine-2-carboxylate | 0.4628 | 0.1584 to 0.6868 | 0.0034 |
|  | 7-methylguanine | -0.3336 | -0.5967 to -0.005813 | 0.0407 |
|  | adenosine 5'-diphosphoribose (ADP-ribose) | -0.3203 | -0.587 to 0.009114 | 0.0499 |
|  | arabonate/xylonate | -0.3428 | -0.6033 to -0.01619 | 0.0351 |
|  | arachidoylcarnitine (C20) | -0.377 | -0.6277 to -0.05536 | 0.0197 |
|  | C-glycosyltryptophan | -0.4017 | -0.645 to -0.08437 | 0.0124 |
|  | cysteine-glutathione disulfide | -0.3566 | -0.6132 to -0.03189 | 0.028 |
|  | eicosanodioate | -0.4536 | -0.6806 to -0.147 | 0.0042 |
|  | eicosenoylcarnitine (C20:1) | -0.3975 | -0.6421 to -0.07945 | 0.0135 |
|  | erythronate | -0.4435 | -0.6738 to -0.1346 | 0.0053 |
|  | inosine 5'-monophosphate (IMP) | -0.3203 | -0.587 to 0.009114 | 0.0499 |
|  | methionine sulfone | -0.3501 | -0.6086 to -0.02448 | 0.0312 |
|  | N4-acetylcytidine | -0.3203 | -0.587 to 0.009114 | 0.0499 |
|  | N6-carbamoylthreonyladenosine | -0.3685 | -0.6217 to -0.04557 | 0.0228 |
|  | N6-succinyladenosine | -0.422 | -0.6591 to -0.1086 | 0.0083 |
|  | N-acetylneuraminate | -0.3654 | -0.6195 to -0.04194 | 0.0241 |
|  | orotidine | -0.5319 | -0.7324 to -0.2465 | 0.0006 |
|  | picolinate | 0.3345 | 0.006799 to 0.5973 | 0.0401 |
